# Exploration and morphological variation of liberoid coffee (*Coffea liberica*) germplasm as a basis for genetic resource conservation

**DOI:** 10.1186/s12870-026-08637-0

**Published:** 2026-04-09

**Authors:** Abdillah Azzam Wahyudin, Yudithia Maxiselly, Intan Ratna Dewi Anjarsari, Citra Bakti, Yani Maharani, Dwi Novanda Sari, Zar Ni Zaw, Haris Maulana

**Affiliations:** 1 Agronomy Magister Program, Faculty of Agriculture, Universitas Padjadjaran, Jl. Raya Bandung- Sumedang Km. 21, Jatinangor, Sumedang, West Java 45363 Indonesia; 2 Department of Agronomy, Faculty of Agriculture, Universitas Padjadjaran, Jl. Raya Bandung- Sumedang Km. 21, Jatinangor, Sumedang, West Java 45363 Indonesia; 3 Department of Plant Pest and Diseases, Faculty of Agriculture, Universitas Padjadjaran, Jl. Raya Bandung-Sumedang Km. 21, Jatinangor, Sumedang, West Java 45363 Indonesia; 4 Agrotechnopreuner Study Program, Faculty of Agriculture, Universitas Padjadjaran, Jl. Raya Bandung-Sumedang Km. 21, Jatinangor, Sumedang, West Java 45363 Indonesia; 5 Myanmar Rubber Planters and Producers Association, Yangon, Myanmar; 6 Research Center for Horticulture, Research Organization for Agriculture and Food, National Research and Innovation Agency (BRIN), Jl. Raya Jakarta – Bogor km 46, Cibinong, Bogor Regency, Bogor, West Java Province 16911 Indonesia

**Keywords:** *Coffea liberica*, Diversity, Exploration, Germplasm, Multivariate analysis, Morphological differences

## Abstract

Liberoid coffee (*Coffea liberica*), a type of coffee cultivated in certain regions in Indonesia. West Java is one of the Indonesian provinces that cultivates liberoid coffee, include Liberica and Excelsa, with limited diversity records. This study aimed to assess the current status of Liberoid coffee in West Java, Indonesia, estimate the morphological diversity, identify the best accession based on morphological traits. An exploratory method was conducted from July to November 2025 to observing Liberoid plantation in Sukabumi, Sumedang, and Kuningan, West Java. Seventy-four accessions were were analyzed using the variance for quantitative characters, correlation, Principal Component Analysis (PCA), cluster analysis, and the multitrait genotype-ideotype distance index (MGIDI). Agronomic practices in each location gave various information such as plant source, field status, and plant maintenance. The variance demonstrated a narrow variance among quantitative characters. The generative morphological traits showed significant correlations with field altitude. The PCA results show seven main components with eigenvalues greater than 1 explained a total variation of 71.216% and Biplot graph from PC1 and PC2 can distinguish across two types of liberoids in various fields. The cluster analysis also explained the distribution patterns of each accession and revealed three distinct groups. The MGIDI index resulted in 11 selected accessions from all observed accessions. These findings could potentially be used as basic for gerplasm conservation of Liberoid coffee, especially in West Java, Indonesia.

**Supplementary Information:**

The online version contains supplementary material available at 10.1186/s12870-026-08637-0.

## Introduction

There are several species of the genus Coffea found worldwide. Only Arabica coffee (*Coffea arabica* L.) and Robusta coffee (*Coffea canephora* Pierre ex A. Froehner) play a significant commercial role in the worldwide coffee industry. Liberoid coffee (*Coffea liberica* Bull. Ex Hiern) contributes to 2% of the world’s total coffee production, whereas other species fail to contribute significantly to production and are poorly known [1, 2]. Indonesia is remarked as one of the producers of Liberoid coffee, which is cultivated across Sumatra, Kalimantan, and Java Islands [3]. Historically, numerous variants of liberoid coffee have been extensively examined in Indonesia [4]. Nevertheless, insufficient documentation hampers the current detection of Liberoid coffee spread in Indonesia.

There are two common variants of Liberica coffee in West Java, namely Liberica (*C. liberica* var. *liberica*) and excelsa (*C. Liberica* var. *dewevrei*). Liberoid coffee has distinct varieties with various morphologies [5]. In general, seed size morphology may be used to distinguish between these two variants [6, 7]. The grouping of liberoid coffee beans is often mixed with other coffee species in the Communities [8, 9]. Both liberica and excelsa types are cultivated mostly in smallholder communities with limited production, and farmers tend to maintain the older liberoid coffees. Previous long-term introduction of liberoid coffee has led to adaptability across various regions in a variety of environments, including peatlands and lowlands, and is resistant to pests and diseases [10, 11].

The observation of potential coffee germplasm can be ulitilzed by exploratory program. Exploration is a way to get plant material by looking at it directly in the field location [12, 13]. Several exploratory research of coffee species has been recorded in Indonesia. Mukhoyyaroh et al. has been investigated leaf characters and number of flower liberica coffee from several agroforestry plantations in Kalipuro, East Java [5, 14]. Wafaretta et al. investigated leaf and tree architecturee of liberica coffee in Poncokusumo, East Java [12]. Exploratory coffee species in Manglayang highland, Sumedang, West Java had obtained one liberica variety, such as LIM 2, and nine arabica varieties [15]. Through phenotypic variance, morphological characters of liberica separated from arabica groups. Morphological identification from previous investigations remains restricted to some plant organs, thus a comprehensive examination of overall morphology to classify liberoid coffee in West Java is required to provide a foundation for future research.

Phenotypic variation can be demonstrated through descriptive statistics and multivariate analysis. Descriptive statistics present data in general terms, including minimum, maximum, standard deviation, and variance. These data describe the distribution of morphological traits and allow inference about the magnitude of phenotypic variation [15, 16]. Multivariate analysis approaches such as Principal Component Analysis (PCA) and Cluster Analysis can project the morphological diversity and distribution patterns from various locations [13, 17]. Meanwhile, the multitrait genotype-ideotype distance index (MGIDI) analysis can identify the best accessions based on observed morphological characters [18–20]. As a result, this research aims to assess the current status, clarify morphological characters, estimate morphological diversity, and identify the best Liberoid coffee accession in West Java using various multivariate analyses.

## Materials and methods

### Study area

**Fig. 1:**
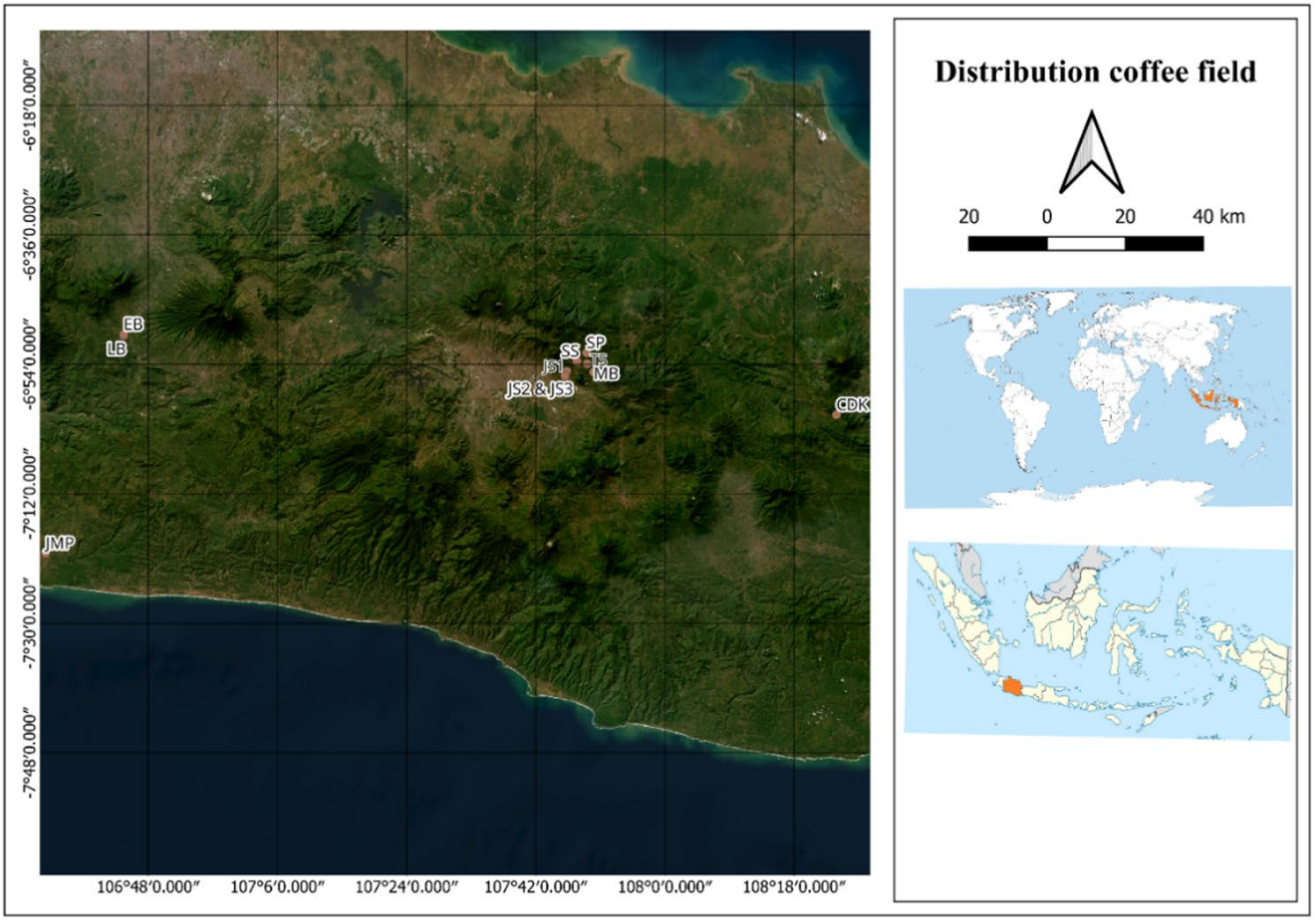
Map of exploration field in West Java. Note: LB = Liberica Balittri, Sukabumi; EB = Excelsa Balittri, Sukabumi; JMP = Jampang, Sukabumi; SP = Sukawangi, Pamulihan, Sumedang; JS = Jatinangor, Sumedang; TS = Tanjungsari, Sumedang; SS = Sukabakti, Sukasari, Sumedang; CDK = Cipasung, Darma, Kuningan; MB = Mekarbakti, Sumedang

The research used the exploratory method with purposive sampling. The method consisting of 2 stages of activity, including exploration or field survey and identification of morphological characters [21]. The research was conducted from July to November 2025 at several locations in West Java, Indonesia, such as Sukabumi, Sumedang, and Kuningan. Accessions from each location are generally classified into two groups, namely in-situ liberoid coffee fields and collection fields. The coordinate map of various observation locations is shown in Fig. 1.

### Materials

The research material is liberoid coffee accessions explored from various observation locations. The tools used included a Global Positioning System (GPS), a measuring tape, meters, and a vernier caliper with an accuracy of 0.02 mm. A questionnaire was used to collect information on agronomic practices from Liberoid coffee plantation maintainers/farmers.

### Procedure

The field survey stage consists of several activities, there are geographical and agronomic data. The geographical data collected including longitude and latitude, which are visualized in Fig. 1 and altitude of origin field. Meanwhile, the agronomic data was obtained through interviews using questionnaires with the information in the form of plant sources, field status, and plant maintenance. The data on the specifics of liberoid coffee plantations in each district were collected from farmers or field maintainers, who served as key informants. This selection was supported by direct field observations in West Java, Indonesia.

Purposive sampling targeted accessions with the sample criteria were healthy coffee plants in the productive stage. The sample size per field location is 10 plants. However, several locations had a limited number of Liberoid trees that met the required criteria during our observation period. Therefore, we considered observing all plants that met the standards in a limited population in certain locations. The number Total 74 selected accessions were labeled with specific codes. The alphabetized codes describe the location of the coffee plantations observed, for example, Jampang and Mekarbakti, abbreviated as JMP and MB, respectively. After the accession origin code, the numbering indicates the accession number observed at each location. Following up to labeling, the stage of morphological character identification can start.

A total of 24 morphological characters of coffee were observed based on the International Plant Genetic Resources Institute (IPGRI) reference and technical observations of coffee bean size, as referred to by the International Union for the Protection of New Varieties of Plants (UPOV) [22, 23]. Qualitative characters consisting of 13 characters, quantitative characters for leaf, fruit, and seed consisting of 7 characters, and flower characteristics consisting of 4 characters were observed if they were found during the same observation period. A score of zero (0) was used when there were no flower morphological characters at the observation location. Table 1 explains the procedure for each character.

**Table 1:** Observation parameters

Characters	Procedure
Habit of plant (HoP)	(1) Bush, (2) Shrub, (3) Tree
Overall appearance (OA)	(1) Elongated conical, (2) Pyramidal, (3) Bushy
Branching habit (BH)	(1) fewer primary branches, (2) Many primary branches with few secondary branches, (3) Many primary branches with many secondary branches, (4) Many primary branches with many secondary and tertiary branches
Angle of insertion of primary branches (AB)	(1) Drooping, (2) Horizontal, (3) Semi-erect
Stipule form (SF)	(1) Roundish, (2) Ovate, (3) Triangular, (4) Deltate, (5) Trapeziform, (6) Other
Young leaf colour (YLC)	(1) Greenish, (2) Green, (3) Brownish, (4) Redish brown. (5) Bronze, (6) Other
Leaf shape (LS)	(1) Obovate, (2) Ovate, (3) Elips, (4) Lanceolate, (5) Other
Leaf apex shape (LAS)	(1) Round, (2) Obtuse, (3) Acute (< 90°), (4) Acuminate, (5) Apiculate, (6) Spatulate, (7) Other
Leaf petiole colour (LPC)	(1) Green, (2) Dark Brown, (3) Other
Young shoot colour (YSC)	(1) Green, (2) Dark brown, (3) Other
Fruit colour (FC)	(1) Yellow, (2) Yellow-orange, (3) Orange, (4) Orange-red, (5) Red, (6) Red-Purple, (7) Purple, (8) Purple-violet, (9) Violet, (10) Black, (11) Other
Fruit shape (FS)	(1) Roundish, (2) Obovate, (3) Ovate, (4) Elips, (5) Oblong, (6) Other
Seed shape (SoS)	(1) Roundish, (2) Obovate, (3) Ovate, (4) Elips, (5) Oblong, (6) Other
Leaf length (LL)	Average of 5 mature leaves at longest part (SI unit: mm)
Leaf width (LW)	Average of 5 mature leaves measured at widest part (SI unit: mm)
Fruit length (FL)	Average of 5 mature fruits measured at the largest part (SI unit: mm)
Fruit width (FW)	Average of 5 mature fruits measured at the widest part (SI unit: mm)
Seed length (SL)	Average of 5 mature seeds (SI unit: mm)
Seed width (SW)	Average of 5 mature seeds (SI unit: mm)
Seed thickness (ST)	Average of 5 mature seeds (SI unit: mm)
Inflorescence on old wood (IO)	(0) Absent, (1) Present
Number of flowers per axil (NF)	Average of 10 axil, if there is no blooming flower part then null (0)
Number of petals (NP)	Average 10 flowers, if there is no blooming flower part then null (0)
Number of stamens (NS)	Average 10 flowers, if there is no blooming flower part then null (0)

Source: IPGRI and UPOV [22, 23]

### Data analysis

The observation data were analyzed using descriptive statistics, heatmap correlation matrix, principal component analysis (PCA), cluster analysis, and the multitrait genotype-ideotype distance index (MGIDI). The presented descriptive statistics include values for minimum (Min), maximum (Max), standard deviation (SD), and variance (σ^2^). Phenotypic variance was used as a measure of trait variability, with two-digit numbers multiplied by the root of the standard deviation of each phenotypic trait. The criteria σ^2^ ≥ 2 × SD σ^2^ indicates state-wide variability, whereas a negative value indicates narrow variability [15, 24].

The heatmap correlation matrix were used to assess the relationships between morphological characters and their relationship to altitude. Correlation were calculated using the Pearson and Spearman correlation coefficients and the criteria for the correlation coefficient as stated in Table 2 [25]. The correlation matrix and its visualization were analyzed using R V4.5.2 with the ggcorrplot package [26].

**Table 2:** Criteria of correlation coefficient values

Range		Level of Correlation
Positive sign	Negative sign	
0.80 to 1.00	-0.80 to -1.00	Very strong
0.60 to 0.79	-0.60 to -0.79	Strong
0.40 to 0.59	-0.40 to -0.59	Moderate
0.20 to 0.39	-0.20 to -0.39	Weak
0.00 to 0.19	-0.00 to -0.19	Very weak

Principal Component Analysis (PCA) and cluster analysis were employed to examine the distribution patterns of accessions. PCA type was performed based on the Pearson correlation matrix. The PCA data results showed the eigenvalues, variations, and factor loadings. Meanwhile, cluster analysis was performed using the Ward’s method and Euclidean distance to find proximity based on dissimilarity values. The datas were processed using Microsoft excel with add-ins XLSTAT 2014 and the visualization of Biplot PCA using R V4.5.2 with multiple packages, such as Factoextra, ggplot2, and ggrepel.

The average and range of variation of 10 quantitative characters were calculated for each clusters from the cluster analysis. Then, Multivariate Analysis of Variance (MANOVA) was employed to assess significant differences among the clusters. Wilks’ lambda and Pillai’s trace were computed with this approach with confidence interval for this test was set at 95%. A number of separate one-way analyses of variance (ANOVAs) were employed to examine the variation among groups for each characteristic. Tukey’s test was used to compare the means of quantitative attributes, with a significance level of α = 0.05 [27]. These analysis was performed using the software IBM SPSS Statistic version 27.

The MGIDI data displayed include selection differentials for each factor analysis, ranking 15% of total accessions, and strengths and weaknesses. A selection rate of around 15% is taken into account when using MGIDI as a selection process and calculating the MGIDI ratio for the i-th accession demonstrates the strengths and weaknesses of each accession [28]. The MGIDI analysis was processed using R Language V 4.5.2 with the metan package [29].

## Results

### Current status of liberoid coffee cultivation in West Java

The Liberoid coffee investigation in this study obtained 74 accessions dispersed throughout three areas in West Java, Indonesia (Table 3). Sukabumi, Sumedang, and Kuningan were the sources of these accessions. Sukabumi had 20 accessions, 18 of which were Liberica and 2 were Excelsa. Furthermore, Sumedang Regency provided 41 excelsa and 3 liberica coffee accessions. Additionally, 10 Kuningan coffee accessions were of the excelsa type. Cultivation techniques vary by location; some fields are maintained regularly, while others are maintained irregularly. Detailed information regarding cultivation techniques at each location is presented in Table 3.

**Table 3:** Distribution of 74 accessions

Accessions	Liberoid type	Origin of Accession (Altitude)	Agronomic Practice
LB1 to LB10	Liberica	The collection of Industrial and Beverage Crop Research Institute, Sukabumi (490 masl)	Clonal material from Liberoid meranti clone series (LIM 1 and LIM 2), collection field with plant maintenance regularly such as fertilization and pest & disease control
LB11	Liberica	The collection of Industrial and Beverage Crop Research Institute, Sukabumi (479 masl)	Liberica collection code: Liberika S19, collection field with plant maintenance regularly such as fertilization and pest & disease control
EB1 & EB2	Excelsa	The collection of Industrial and Beverage Crop Research Institute, Sukabumi (480 masl)	Collection field with plant maintenance regularly such as pruning, fertilization, and pest & disease control
JMP1 to JMP7	Liberica	Jampang District, Sukabumi (136 masl)	Farmer-maintained population, old coffee field without pruning. Weeding iregularly
JS1	Liberica	Jatinangor District, Sumedang (790 masl)	Clonal material, collection field with plant maintenance regularly such as pruning, fertilization, and pest & disease control
JS2 & JS3	Liberica	Jatinangor District, Sumedang (790 masl)	Collection field (Collected from Kalimantan), maintenance without pruning and weeding regularly
SP1 to SP20	Excelsa	Two different fields (10 accessions per field), Sukawangi Village, Pamulihan District, Sumedang (950 masl)	Farmer-maintained population, old coffee field near residential area without pruning. Plant maintenance regularly such as fertilization and plant & diseases control.
TS1 to TS7	Excelsa	Gunungmanik, Tanjungsari District, Sumedang (870 masl)	Farmer-maintained population, old coffee field with plant maintenance such as pruning and weeding.
SS1 to SS10	Excelsa	Sukabakti Village, Sukasari District, Sumedang (900 masl)	Farmer-maintained population from bare-root seedling, coffee field near residential area with fertilization regularly.
CDK1 to CDK10	Excelsa	Cipasung Village, Darma District, Kuningan (760 masl)	Farmer-maintained population, coffee field near residential area with plant maintenance regularly such as pruning, fertilization, and pest & disease control.
MB1 to MB4	Excelsa	Mekarbakti Village, Pamulihan District, Sumedang (900 masl)	Farmer-maintained population, old coffee field near residential area without pruning and weeding iregularly. Excelsa coffee as shading plants.

*Abbreviation*: *LB* Liberica Balittri, *EB* Excelsa Balittri, *JMP* Jampang, *SP* Sukawangi, Pamulihan, *JS* Jatinangor, Sumedang, *TS* Tanjungsari, Sumedang, *SS* Sukabakti, Sukasari, *CDK* Cipasung, Darma, Kuningan, *MB* Mekarbakti, *Masl*  meters above sea level

### Descriptive statistic

Variability is a fundamental tool for measuring the composition of accession populations. Table 4 presents the phenotypic variance for all accessions across morphological characters. The data demonstrate all quantitative characters have a narrow variation (σ^2^ ≤ 2 × SD σ^2^).

**Table 4:** Descriptive statistics and variance of quantitative characters

Traits	Min	Mean	Max	SD	σ^2^	2 × SD	Note
Leaf length	100	249.90	350	40.69	12.76	81.38	Narrow
Leaf width	67.80	235.00	126.12	28.39	10.66	56.77	Narrow
Fruit length	10.43	16.93	28	3.57	3.78	7.14	Narrow
Fruit width	9.2	14.28	22	2.66	3.26	5.33	Narrow
Seed length	8.6	12.49	19.2	2.61	3.23	5.23	Narrow
Seed width	5.10	16.20	8.82	2.14	2.93	4.29	Narrow
Seed thickness	3.50	19.10	7.37	4.46	4.22	8.92	Narrow
Number of flowers per axil	0	1.52	11.6	2.94	3.43	5.88	Narrow
Number of petals	0	0.73	6.57	1.84	2.71	3.68	Narrow
Number of stamens	0	0.63	6.5	1.63	2.55	3.25	Narrow

*Min* Minimum, *Max* Maximum, *SD* Standard deviation, *σ*^2^  Variance

### Correlation altitude and the morphological characters of liberoid coffee

**Fig. 2:**
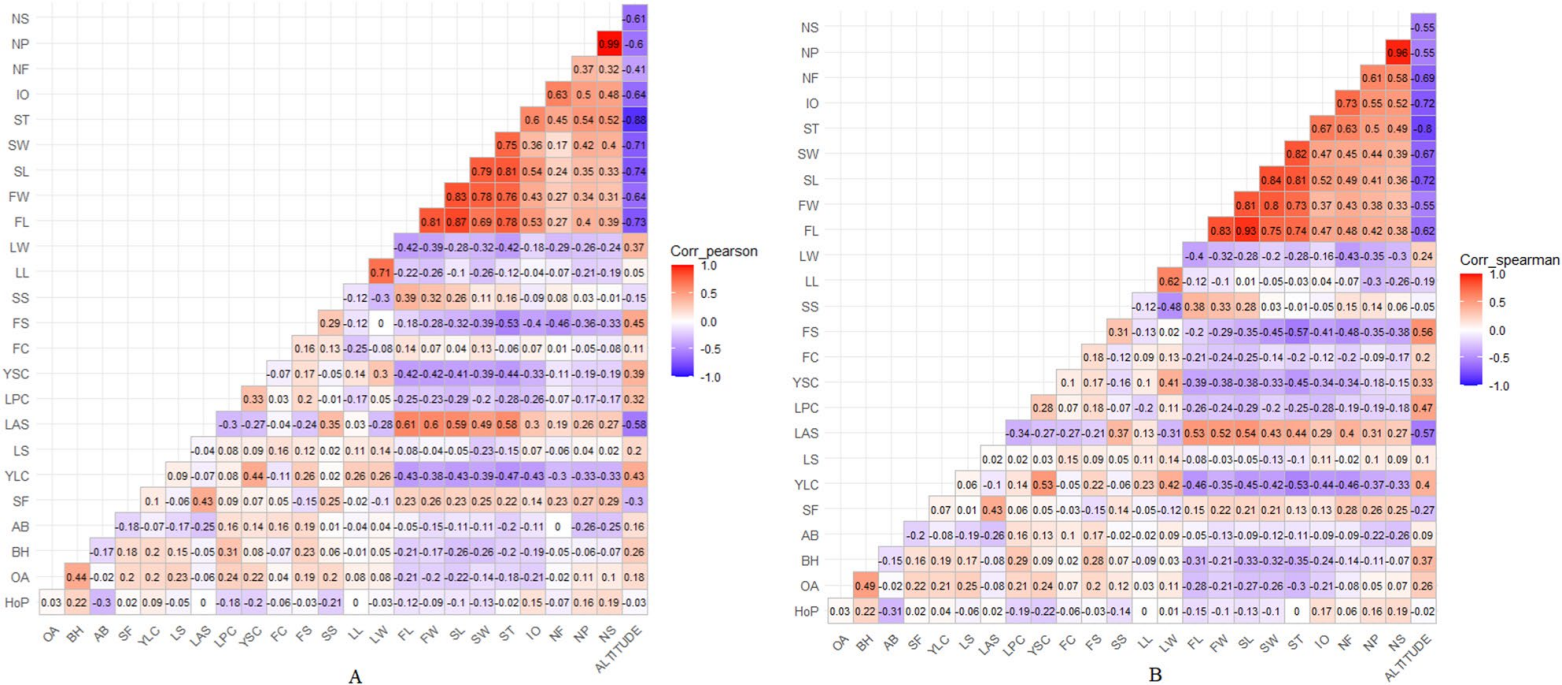
Heatmap correlation matrix of altitude and morphological characters. **A** Pearson Correlation, **B** Spearman Correlation

This study found that liberoid coffee in West Java, grown at diverse altitudes, exhibits numerous morphological variations. Figure 2 shows the correlation matrix of altitudes and their morphological characteristics using pearson and spearman correlations. The Pearson Correlation analysis results indicate that most morphological traits have a weak to moderate relationship with altitude, with some negative correlations for reproductive variables such as flower, petal, and stamen number. On the other hand, vegetative and fruit size traits tend to show more variable correlations, but not all are significant or strong. This pattern indicates that the relationship between altitude and morphology is not entirely linear and may be influenced by other factors. Furthermore, the inconsistent variation in correlation values ​​between variables also suggests that the linearity assumption of Pearson correlation may not always be met for all data.

To address these limitations, the analysis was supplemented with Spearman Rank Correlation, which does not rely on the assumptions of normality and linearity. The Spearman Rank Correlation results show a pattern that is generally consistent with Pearson correlation, particularly regarding the direction of the relationship (positive/negative), although with slightly different correlation strengths. This consistency confirms that the findings of the relationship between altitude and several morphological traits are quite robust, although not always strong. However, both approaches also indicate that altitude is not the sole determinant of morphological variation, so the possibility of confounding factors such as differences in agronomic practices between locations still needs to be considered in interpreting the results.

### Principal component analysis

PCA of the morphological traits results seven principal components (PCs) with eigenvalues greater than 1, accounting for a cumulative 71.889% of the total variation among the 74 Liberoid coffee accessions, as detailed in Table 5. This suggests a high degree of morphological diversity in the coffee accessions studied. The first principal component (PC1) accounted for 29.62% of the variation. It was primarily influenced by YLC, LAS, YSC, FL, FW, SL, SW, ST, IO, NP, and NS. The second principal component (PC2) contributed 9.578% of the variation and was influenced by NS. PC3 explained 9.267% of the variation, mainly affected by OA, BH, SF, and SS. The fourth principal component (PC4) accounted for 7.523% and was influenced by LAS LL, while PC5 accounted for 6.323% and was influenced by HoP and YSC. PC6 contributed 5.306% influenced by LS and FC; and PC7 explained 4.272% with the influence by NF. Generally, quantitative traits contributed more significantly to the observed morphological diversity among the 74 Liberoid coffee accessions as detailed in Table 5, with each presenting a unique coefficient value.

**Table 5:** Eigenvalue, variabilty, total variation, and loading factor in each PC

	PC1	PC2	PC3	PC4	PC5	PC6	PC7
Eigenvalue	7.109	2.299	2.224	1.806	1.517	1.273	1.025
Variability (%)	29.620	9.578	9.267	7.523	6.323	5.306	4.272
Cumulative %	29.620	39.198	48.465	55.988	62.311	67.617	71.889
Loading factor							
Habit of plant	0.000	0.483	0.047	-0.118	**-0.637**	-0.001	0.205
Overall appearance	-0.221	0.191	**0.670**	0.001	0.171	0.133	-0.064
Branching habit	-0.267	0.206	**0.611**	-0.019	-0.175	-0.012	0.323
Angle of insertion of primary branches	-0.205	-0.462	-0.218	-0.217	0.440	-0.020	0.055
Stipule form	0.304	0.150	**0.550**	0.251	0.216	-0.255	0.088
Young leaf colour	**-0.547**	0.099	0.253	0.414	-0.091	-0.178	-0.020
Leaf shape	-0.127	0.158	0.260	0.059	0.069	**0.759**	-0.060
Leaf apex shape	**0.639**	-0.048	0.233	0.496	-0.103	-0.084	0.086
Leaf petiole colour	-0.348	-0.139	0.367	-0.285	0.359	-0.154	0.017
Young shoot colour	**-0.524**	0.073	0.200	0.077	0.471	-0.185	-0.212
Fruit colour	0.056	-0.371	0.121	-0.233	0.057	**0.524**	0.044
Fruit shape	-0.481	-0.456	0.337	-0.050	-0.270	0.196	-0.096
Seed shape	0.221	-0.472	**0.530**	0.246	0.016	0.042	0.091
Leaf length	-0.264	0.386	-0.253	**0.677**	0.225	0.178	0.078
Leaf width	-0.487	0.330	-0.257	0.446	0.257	0.247	-0.158
Fruit length	**0.859**	-0.291	0.073	0.139	0.001	0.103	-0.019
Fruit width	**0.839**	-0.254	0.076	0.186	-0.043	0.029	-0.018
Seed length	**0.860**	-0.189	-0.053	0.269	0.028	0.091	-0.061
Seed width	**0.806**	-0.176	-0.029	0.088	0.024	-0.102	-0.257
Seed thickness	**0.915**	0.048	-0.042	0.080	0.051	-0.069	0.035
Inflorescence on old wood	**0.679**	0.305	-0.167	-0.146	0.163	0.270	0.305
Number of flowers per axil	0.481	0.244	0.016	-0.227	0.452	-0.040	**0.562**
Number of petals	**0.636**	0.489	0.236	-0.309	0.075	0.024	-0.357
Number of stamens	**0.615**	**0.501**	0.224	-0.298	0.048	0.003	-0.386

Values in bold > 0.5 (Positive sign) or <-0.5 (Negative sign) are contribute to PCs

**Fig. 3:**
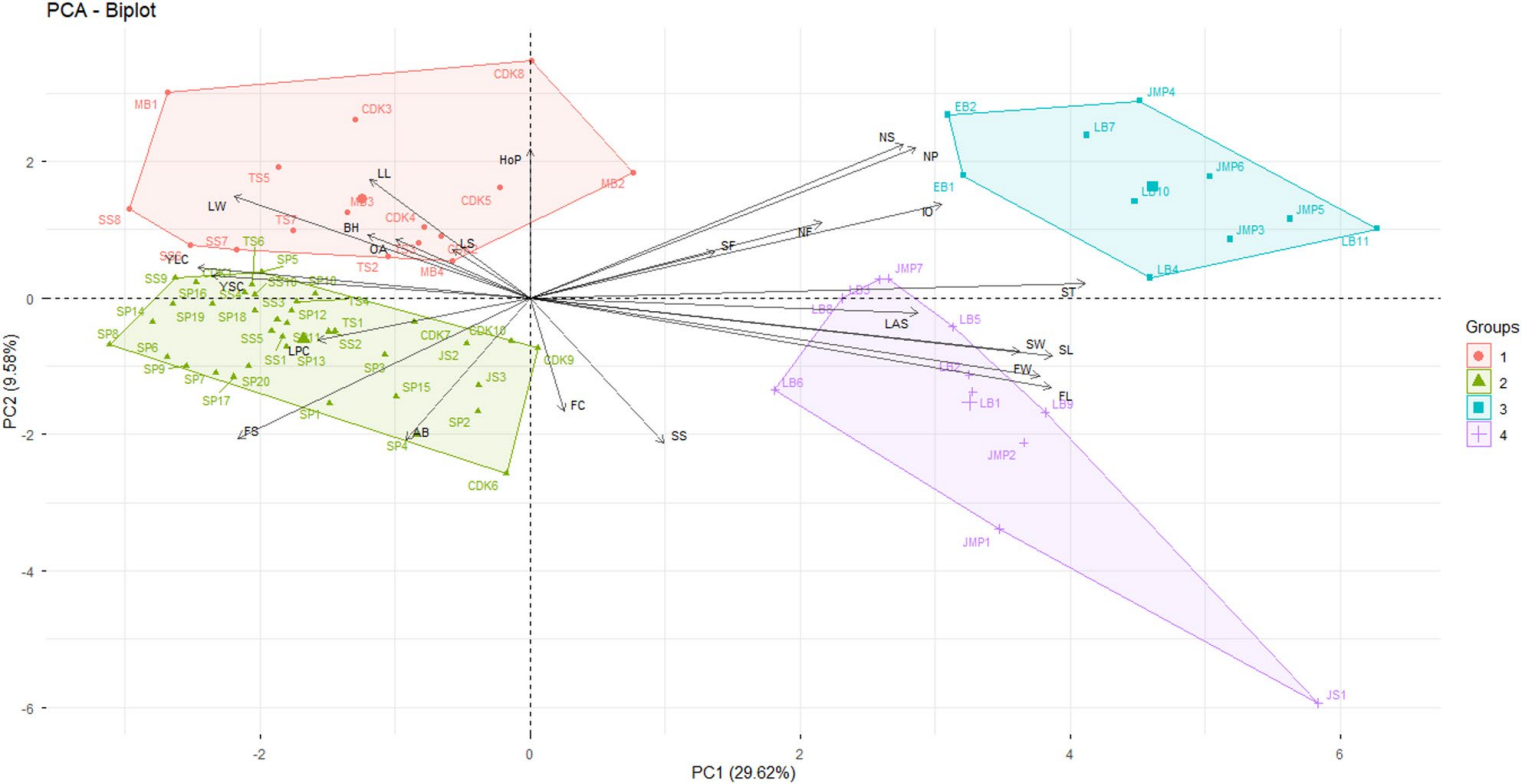
Biplot PCA of 74 Accessions. LB = Liberika Balittri; EB = Excelsa Balittri; JMP = Jampang, Sukabumi; SP = Sukawangi, Pamulihan; JS = Jatinangor, Sumedang; TS = Tanjungsari, Sumedang; SS = Sukabakti, Sukasari; CDK = Cipasung, Darma, Kuningan, MB = Mekarbakti, Sumedang

PCA biplot featuring the distribution of accessions is shown in Fig. 3, capturing 39.2% of the total variation (PC1 = 29.62%; PC2 = 9.58%). The accessions in the PCA biplot were separated into four large groups. Group 1 consisted of accessions MB (MB1-MB4), TS2, TS3, TS5, TS7, SS6-SS8, CDK2-CDK5, and CDK8. Group 3 consisted of accessions JMP3-JMP6, LB4, LB7, LB10, LB11, EB1, and EB2. Group 4 included JS1, JMP1, JMP2, JMP7, LB1-LB3, LB5, LB6, LB8, and LB9. The remaining accessions were included in group 2. Based on the comparison of each accession, accession JS1 was distinct from the other accessions, while SS3 and SS5 clustered closely, reflecting high similarity. The following are other tightly grouped accessions, such as TS4 and SS10; TS6 and SS3; LB1, LB2, and LB5. A large cluster including excelsa accessions (CDK, MB, SS, SP, and TS) and 2 liberica accessions (JS2 and JS3).

The direction of the morphological character vector determines the links between accession groups and specific morphological characters. The closer an accession is to a particular character, the higher the character in that accession. HoP, BH, OA, LL, LW, and LS are related to Group 1; Group 2 has a strong influence on characters AB, FS, and LPC, notably SP4 with AB character; EB1 and EB2 in group 3 are adjacent to the NP and NS characters; LB9 in group 4 has a strong influence on FL and FW characters.

Correlation analysis based on angle vector relationship showed significant positive relationships among several traits. Character leaf length was positively correlated with leaf width and the habit of the plant. Young leaf colour showed a significant correlation with young shoot colour and leaf petiole colour. Fruit size (FL and FW) and seed size characters (SL, SW, and ST) showed positive correlations with each other. Likewise, inflorescences on old wood were significantly correlated with the number of petals, flowers, and stamens.

Visual interpretation of the PCA biplot is exploratory in nature and needs to be supported by inferential testing. The PCA biplot provides an initial indication of a pattern of separation between groups, but cannot be used as strong statistical evidence. Therefore, to strengthen the claim of differences between Liberica and Excelsa, the analysis was supplemented with a Multivariate Analysis of Variance (MANOVA) test. The MANOVA results indicated significant differences between groups based on the combination of analyzed variables (*p* < 0.001), thus statistically supporting the separation previously observed in the PCA biplot (Table 6). This finding indicates that variation between groups significantly affects the overall dependent variable. Therefore, further analysis (univariate or post hoc test) is necessary to identify which variables contribute most to these differences.

**Table 6:** Manova result using different statistical test

Test	Value	F	Hypothesis df (df1)	Error df (df2)	Sig.
Pillai’s Trace	1.028	6.659	20	126	< 0.001
Wilks’ Lambda	0.087	14.769	20	124	< 0.001

The results of Tukey’s post hoc test showed that most variables were able to differentiate clusters significantly, indicated by different letters in each cluster (Table 7). Cluster 1 generally had the highest values, especially for leaf length, fruit length, fruit width, seed length, seed width, and seed thickness, and were significantly different from the other clusters. Cluster 2 tended to be in the middle position, but for several variables such as seed width and thickness, it showed the lowest values. Meanwhile, cluster 3 generally had the lowest values, especially for reproductive characters such as number of petals, number of flowers per axil, and number of stamens, which were significantly different from the other two clusters. However, there were variables that did not show significant differences between clusters, such as leaf length (*p* = 0.092). Overall, these results confirm that cluster separation is supported by significant differences in most morphological characters, especially in the size of fruit, seeds, and reproductive organs.

**Table 7:** Average of quantitave characters for each cluster from cluster analysis of the *coffea liberica*

	Cluster 1 (*n* = 22)	Cluster 2 (*n* = 24)	Cluster 3 (*n* = 28)	F (Anova)	Sig
Leaf length (LL)	237.94^a^	246.49^a^	262.21^a^	2.404	0.092
Leaf width (LW)	103.59^a^	130.81^b^	139.79^b^	14.343	< 0.001
Fruit length (FL)	21.25^b^	15.01^a^	15.17^a^	60.487	< 0.001
Fruit width (FW)	17.46^b^	12.81^a^	13.04^a^	56.191	< 0.001
Seed length (SL)	15.60^b^	11.20^a^	11.15^a^	54.952	< 0.001
Seed width (SW)	11.15^b^	7.95^a^	7.72^a^	36.879	< 0.001
Seed thickness (ST)	13.64^b^	5.10^a^	4.39^a^	206.814	< 0.001
Number of petals (NP)	2.23^b^	0.21^a^	0^a^	14.416	< 0.001
Number of flowers per axil (NF)	3.63^b^	1.36^a^	0^a^	12.426	< 0.001
Number of stamens (NS)	1.89^b^	0.2^a^	0^a^	12.395	< 0.001

Different letters denote significant differences among clusters (Tukey post-hoc test, *p* = 0.5)

### Cluster analysis

**Fig. 4:**
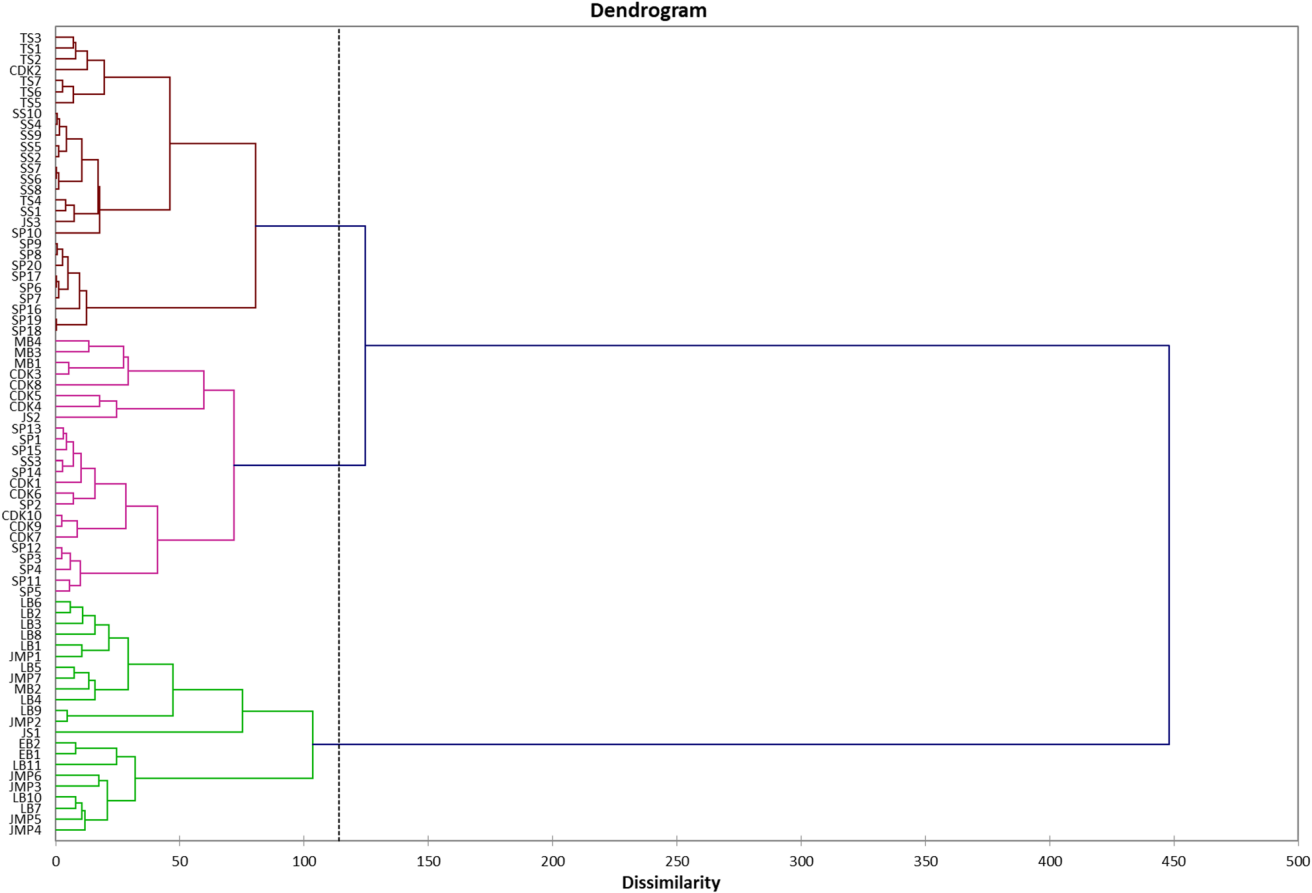
Cluster analysis of 74 accessions

The grouping of liberoid coffee accessions is shown in the dendrogram (Fig. 4). A total of 74 accessions were grouped using Ward’s method, yielding three main groupings. The dissimilarity values ​​from the Ward model can be used in an alternative interpretation. Based on the cutpoint of 113.98, the three main groupings were identified. Group 1 has 22 accessions (JMP1, JMP2, JMP3, JMP4, JMP5, JMP6, JMP7, LB1, LB2, LB3, LB4, LB5, LB6, LB7, LB8, LB9, LB10, EB1, EB2, LB11, JS1, and MB2), Group 2 has 24 accessions (SP1, SP2, SP3, SP4, SP5, SP11, SP12, SP13, SP14, SP15, SS3, CDK1, CDK3, CDK4, CDK5, CDK6, CDK7, CDK8, CDK9, CDK10, JS2, MB1, MB3, and MB4), and Group 3 has 28 accessions. The data show Euclidean distances ranging from 0.743 to 14.209. The smallest Euclidean value is between accessions SP18 and SP19, while the maximum value is between accessions JS1 and MB3.

### Multitrait genotype-ideotype distance index for liberoid coffee

A Multitrait genotype-ideotype distance index (MGIDI) analysis identified the top 15% of accessions and evaluated their morphological characters. In this study, we projected selection differentials into factor analysis (FA), accession ranking, and a strengths-and-weaknesses view of selected accessions. The selection differential for 24 morphological characters into FA groups is shown in Table 8. The factor analysis grouping is determined by eigenvalues greater than 1, yielding 7 factors. FA1 contributes to the characteristics of leaf apex shape, seed shape, fruit size (length and width), and bean size (length, width, and thickness), while FA2 has the characteristics of NP and NS. This interpretation continues for the other FAs. This interpretation continues for the other FAs.

**Table 8:** Selected differentials of accessions from 24 morphological characters

Characters	Factor	Goal	Xo	Xs	Selection differential
Leaf apex shape (LAS)	FA1	100	3.784	4.000	0.216
Seed shape (SS)	FA1	100	3.784	4.000	0.216
Fruit length (FL)	FA1	100	16.926	17.890	0.964
Fruit width (FW)	FA1	100	14.278	15.055	0.778
Seed length (SL)	FA1	100	12.490	13.865	1.375
Seed width (SW)	FA1	100	8.816	9.695	0.878
Seed thickness (ST)	FA1	100	7.371	9.470	2.099
Number of petals (NP)	FA2	100	0.732	2.015	1.284
Number of stamens (NS)	FA2	100	0.628	1.618	0.990
Overall appearance (OA)	FA3	100	2.378	3.000	0.622
Brancing habit (BH)	FA3	100	2.986	3.273	0.286
Stipule form (SF)	FA3	100	2.662	2.909	0.247
Young leaf colour (YLC)	FA3	0	2.743	2.364	-0.380
Leaf length (LL)	FA4	100	249.896	286.455	36.559
Leaf width (LW)	FA4	100	126.116	151.509	25.393
Habit of plant (HoP)	FA5	0	2.189	1.909	-0.280
Angle of insertion of primary branches (AB)	FA5	0	2.095	2.000	-0.095
Leaf petiole colour (LPC)	FA5	0	1.297	1.182	-0.115
Young shoot colour (YSC)	FA5	100	1.743	2.000	0.257
Leaf shape (LS)	FA6	100	2.851	3.091	0.240
Fruit colour (FC)	FA6	100	4.986	5.000	0.014
Fruit shape (FS)	FA7	0	2.959	2.455	-0.505
Inflorescence on old wood (IO)	FA7	100	0.392	0.545	0.154
Number of flowers per axil (NF)	FA7	100	1.522	2.555	1.033

*Xo*  original population mean, *Xs*  the mean of selected accessions

**Fig. 5:**
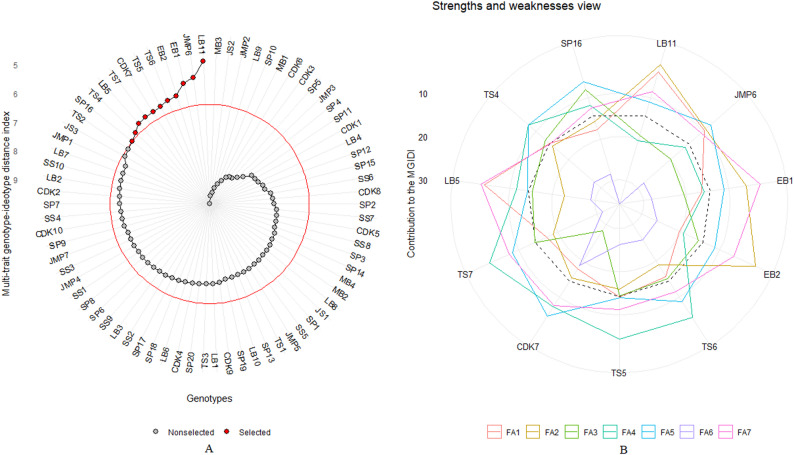
MGIDI Interpretation for selection accession. **A** Accession ranking based on the MGIDI Value, showing the best accessions start from red circle as a cutpoint. **B** Strengths and weaknesses view of the selected accession, the characters near to the inner edge have more contribution of factor

Figure 5A shows the ranking of 74 accessions based on MGIDI index values. Accessions selected based on this index are shown in red, and the red circle in the center indicates the boundary of the entire accession. The selected accessions were 11, in ascending order: LB11, JMP6, EB1, EB2, TS6, TS5, CDK7, TS7, LB5, TS4, and SP16. Next, the 11 accessions were presented with strengths and weaknesses based on the contribution value of each FA to MGIDI (Fig. 5B). In this study, LB11 showed strengths in the related characters in FA1, such as LAS, SS, FL, FW, SL, SW, and ST; but showed weaknesses in the related traits in FA4 and FA6, namely LL and LW in FA4, and LS and FC in FA6. On the other hand, EB2 had a lesser impact on related characters in FA1 (with a high contribution to the MGIDI) but this accession had better performance than the 10 selected accessions in FA2. CDK7 shows the poorest performance for FA3, particularly for the character of overall appearance (OA), brancing habit (BH), stipule form (SF), and young leaf colour (YLC). FA4 has a high contribution ranking to the MGIDI in accession TS7. Furthermore, CDK7 had the highest contributed ranking to FA5 and FA6 among the chosen accessions. Generally, the contribution of FA7 to the MGIDI was small, suggesting that the 11 selected accessions had desired characters in FA7, such as fruit shape (FS), inflorescence on old wood (IO), and number of flowers (NF).

## Discussions

The liberoid coffee plantations were managed with various agronomic practices (Table 3). Liberoid coffee accessions are often in-situ plantations, either cultivated by smallholder farmers with unknown plant ages. The farmers in Sumedang decided not to take down their Excelsa coffee trees and didn’t replace them with other crops. Farmers in Jampang, Sukabumi, have also preserved the old Liberica coffee trees. Despite the fact that the accession in several fields is ancient, the liberoid coffee is still productive. In addition, the Excelsa coffee farmers in Cipasung, Kuningan, have independently selected parent trees as sources for propagation. There are also Liberoid coffee fields that are part of the collection field with under proper maintenance.

Based on this brief description, various liberoid coffee germplasm in West Java is still being preserved using various agronomic practices. Liberoid coffee is planted in certain areas of West Java in mixed fields with other commercial coffee species (arabica and/or robusta). This model field also occurs among farmers in Uganda who maintain mixed Robusta-Excelsa coffee fields [30]. Proper in situ preservation can be an option for obtaining accessions for ex situ conservation and other development, such as agronomic & plant breeding purpose [2].

The difference in phenotypic variance is due to environmental conditions unique to each accession location. Environmental factors that can affect this include adaptability, uncontrolled conditions, and variation in accessions at the same location [16]. These factors could not affect the genotype in the coffee plant [13]. Narrow phenotypic variance on Table 4 tends to indicate genotypic homogeneity. Environmental factors affect vegetative growth, inflorescence, and bean development. The morphology of coffee can be influenced by exogenous factors, such as field altitude. The Pearson correlation analysis shows a significant negative correlation between the altitude of the coffee field and traits related to fruit and seed size (Fig. 2). This indicates that Liberoid coffee produces relatively large fruit and seeds at low altitudes. Altitude contributes to variances in coffee plant quality and shape in Brazil [31]. According to Pangestika et al., field location affects coffee plant morphology and adaptability [12]. Land altitude affects both macro- and microclimates, which, in turn, alter coffee morphological traits [32]. Consequently, flowering did not occur simultaneously across all locations. Several Liberoid accretions found tend to exhibit fluorescence on old wood. This characteristic enables branches that have already produced fruit to continue producing fruit the next season. Arabica coffee shows similar characteristics in inflorescence on old wood, but Liberica coffee possesses plagiotropic branches that can develop orthotropic branches, serving as a differentiator [33].

The vegetative and generative characters of liberoid coffee may be influenced by phenotypic plasticity. Phenotypic plasticity refers to phenotypic variation in response to environmental conditions, especially in climate instability [34, 35]. Morphological characters with no significant correlation with altitude indicating have little phenotypic plasticity. The lack of phenotypic plasticity signifies inadequate adaptation to altitude [12]. Meanwhile, wide phenotypic plasticity demonstrate to be essential under various altitude conditions.

The diversity of accession based on the observation dataset can be explained by the PCA (Table 5 and Fig. 3). This method can reduce the data dimensions to principal components (PC). The PCs from PC1 to PC7 have eigenvalues greater than 1, indicating that the components influence accession groups. Two sizable groups comprise the majority of the accession diversity in Fig. 3. Coffee groups with accession codes from Sukabumi (EB, LB, and JMP) and Liberica Sumedang (JS1) are not closely related to excelsa accessions from Sumedang or Kuningan. In contrast, the excelsa accession codes from Sumedang (SS, SP, TS, MB) and Kuningan (CDK) show a very tight relationship. Furthermore, the Biplot PCA results indicate the presence of separated groups. We found accessions that we consider liberica type, but are included in the excelsa group, namely accessions JS2 and JS3. We discovered accessions that we thought liberica but got placed in the excelsa category in group 2, notably accessions JS2 and JS3. Similarly, excelsa EB2 and EB3 were among the several sukabumi origin in group 3. The overlapping plot PCA findings between Liberica and Excelsa were also evident in different characters, such as pollen and floral metrics [36, 37]. Natural plant characters involved in the overlap of Liberoid coffee [38]. In addition, overlapping may occur due to environmental sensitivity and plant age [39].

Biplot graphs can also be used to evaluate the correlation between characters. Characters that overlap around an angle of 0° show a high positive correlation, but characters that are opposite (> 90°) have a negative correlation [17, 40]. For example, quantitative traits of fruit and seeds show a strong positive correlation, particularly between fruit length and width. Meanwhile, one of the negative correlations that formed an 180° angle was found between fruit length and the colour of young leaves. The dimensions of the fruit and seeds are linked: as the fruit becomes larger, so do the seeds [41]. Besides the correlation of characters in the PCA vector, we show a correlation heatmap (Fig. 2).

The diversity of accessions can be projected using cluster analysis. Accessions with Euclidean distances less than 0.75 indicate small dissimilarities and a near relationship [42, 43]. Both SP18 and SP19 exhibit similar morphological appearances, as indicated by their quantitative and qualitative characteristics (Euclidean distance = 0.743). There are several accessions at the same field site with a large dissimilarity, for example, between CDK5 and CDK8 (Euclidean distance = 8.339). This demonstrates that the same agronomic practice can result in distinct relationship patterns.A large dissimilarity score suggests considerable variations between groups (Fig. 4). Some old coffee trees endured regional extinctions and considerable changes in distribution patterns, yet their genetic diversity was preserved. Even when the parent tree’s genetic origin is unknown, natural selection of old trees can retain or even increase genetic diversity [44]. Genetic diversity in metapopulations of old trees endures despite long-term environmental changes [45]. The geography of the coffee field also contributed to the genetic level [46]. Accessions with high levels of genetic variety are more likely to be used in plant breeding programs because their genetic diversity can yield offspring with superior qualities [47].

The MANOVA results in this study indicate significant multivariate differences between groups, confirming that the combination of dependent variables simultaneously differs between groups. The MANOVA approach is important because it captures the combined effects of multiple variables simultaneously, reducing the risk of Type I errors due to repeated testing [48]. Furthermore, recent research has highlighted more flexible MANOVA methods for high-dimensional data or when the assumption of normality is not met, for example, using the geometric median and bootstrapping [49, 50]. Thus, the multivariate significance obtained not only confirms the findings of patterns of differentiation between groups but is also consistent with modern multivariate analysis practices that are robust to assumption violations.

MGIDI is the latest analytical approach for selecting genotypes or accessions in plant breeding programs. The MGIDI analysis has been used for various crops, including rice mutants, coffee, and wheat [18, 20, 28]. MGIDI was able to project genotypes and evaluate characters using factor analysis (FA) and genotype-ideotype distance [51]. The characters reached the 100% selection goal indicating the effectiveness of MGIDI for considering agronomic characters (Table 8). Barajehfard et al. confirmed the seletion goal results highlight the index ability to prioritize genotype [52]. This approach also facilitates interpretation of the accessions indicated by the MGIDI index.

According to the accession ranking results, 11 liberoid coffee accessions were selected (Fig. 5A). Liberica accession LB11 exhibited the most characteristic features of the entire liberoid group. Meanwhile, EB1 performed the best in the excelsa group when compared to other excelsa accessions. The selected accessions are further characterized using a strengths-and-weaknesses analysis that describes the contribution of each factor in the radar plot (Fig. 5B). The strengths and weaknesses view provided an ideal guidance for selecting accession [53, 54]. The smaller the proportion explained by a factor (the closer it is to the outer edge), the closer the characters in that factor are to the ideal accession [18]. Meanwhile, the cutpoint lines reflect the average performance in factor contribution. If the contribution is below average, this shows the weakness of accession [55]. FA1 had a higher ranking than cutpoint at LB11, JMP6, JS3, TS2, TS4, TS5, EB1, and TS6, indicating that these accessions had better performance of leaf apex shape, seed of shape, fruit length, fruit width, seed length, seed width, and seed thickness. Conversely, TS7, CDK7, and EB2 demonstrated below-average contribution, indicating their weaknesses in FA1. Through this strategy, researchers can target the development of the characteristics they want more accurately [19, 20, 28].

The current finding has limitations, including the limited floral organ seen in liberoid coffee accessions and DNA variations. Plant phenotypic variation is typically identified using fruit and seed characteristics [41, 56, 57]. In fact, floral traits are also critical to differentiate coffee species, including liberoid coffee types [36, 37]. For example, floral characters include corolla shape, flower colour, and number of petals, which should be observed in future research. The Liberica type has up to 10 petals, while the Excelsa type has an average of 5 [37]. Gene-level studies (e.g. molecular marker) may better differentiate liberoid coffee types. This advice can provide additional reasons to identify accessions representing liberoid coffee types and for hybridization purposes in the future. Furthermore, agroecological and yield components should be included to better document the representative of liberoid coffee fields and to capture the interaction between genotype and environment, specifically in West Java, Indonesia. In addition, this study was conducted over a single growing season, which limits the ability to fully separate genetic differences from environmental influences such as altitude, climate, and cultivation practices. Traits like leaf and fruit size, seed dimensions, and reproductive features are particularly sensitive to environmental variation, and genotype-by-environment (G×E) interactions could not be captured. Nevertheless, the results provide an initial overview of phenotypic trends and multivariate patterns among Liberica and Excelsa accessions. Future multi-season trials would allow more accurate separation of genetic and environmental effects, strengthen confidence in genotype evaluation, and better inform both breeding and conservation efforts.

## Conclusions

Based on the explanation above, liberoid coffee has been well maintained in West Java, Indonesia. The Pearson correlation showed that variations in the altitude of the coffee field also influenced morphological characters. Analysis using the phenotypic variance approach obtained a wide range of morphological diversity, generally represented in qualitative characters. The findings from PCA and cluster analysis provide evidence that certain accessions exhibit distinct morphological characters. These approaches also found that there were intersections between different liberoid groups, which showed morphological similarities. Liberica accession JS2 has similarities with the excelsa group. In addition, analysis using the MGIDI approach identified 11 selected accessions from a total of 74. The best accessions using this approach was LB11, JMP6, TS5, TS2, TS7, EB1, TS4, TS6, SP16, CDK7, and JMP1. These results provide a preliminary foundation for understanding liberoid coffee in West Java, supporting further development of agronomic practices and plant breeding.

## Supplementary Information

Supplementary Material 1.

## Data Availability

The data presented in this study are available upon request from the corresponding author.
