# Supplementary material for: Exploration and morphological variation of liberoid coffee (Coffea liberica) germplasm as a basis for genetic resource conservation
Source: BMC Plant Biol. 2026 Apr 9;26:845. doi: 10.1186/s12870-026-08637-0 (PMC13173790; doi:10.1186/s12870-026-08637-0)
Supplement: Supplementary file 1 — Supplementary Material 1. [file 12870_2026_8637_MOESM1_ESM.docx]

| SUKASARI, SUMEDANG ORIGIN | |
| --- | --- |
| SS1 | SS2 |
| 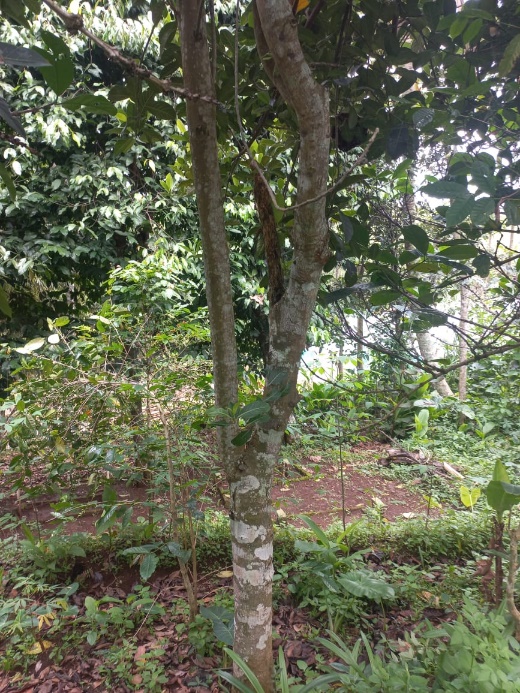 | 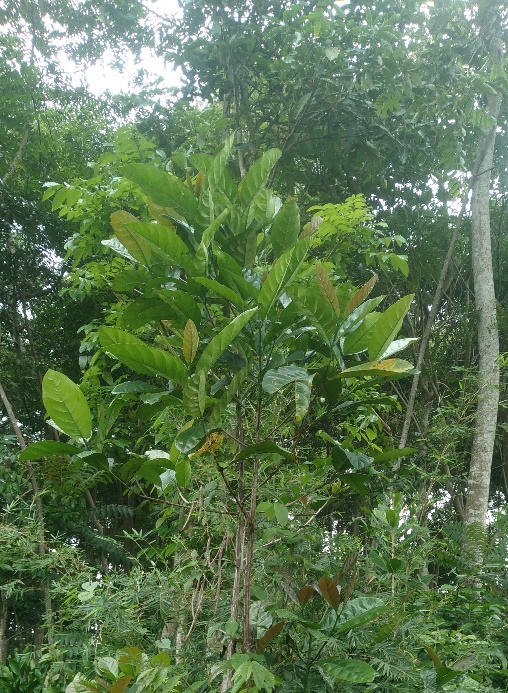 |
| SS3 | SS4 |
| 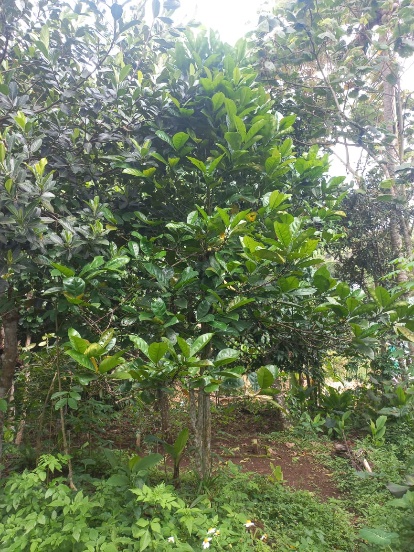 | 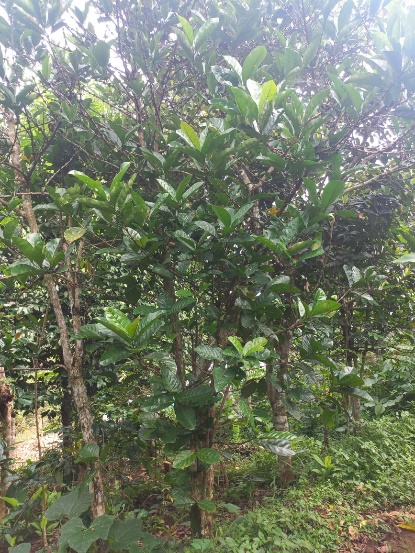 |
| SS5 | SS6 |
| **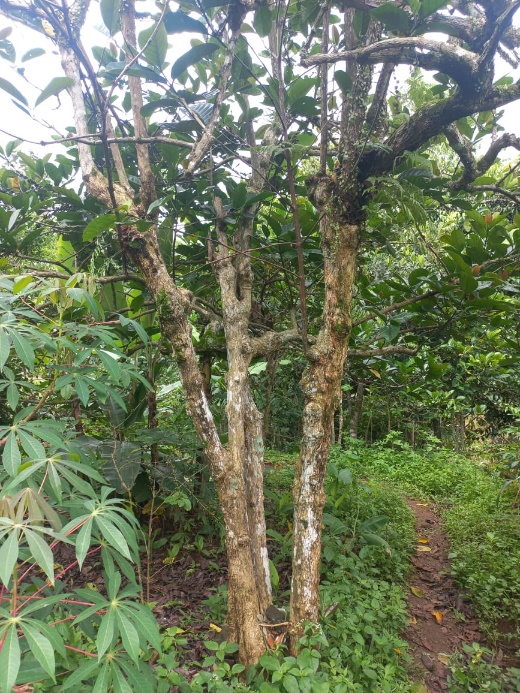** | 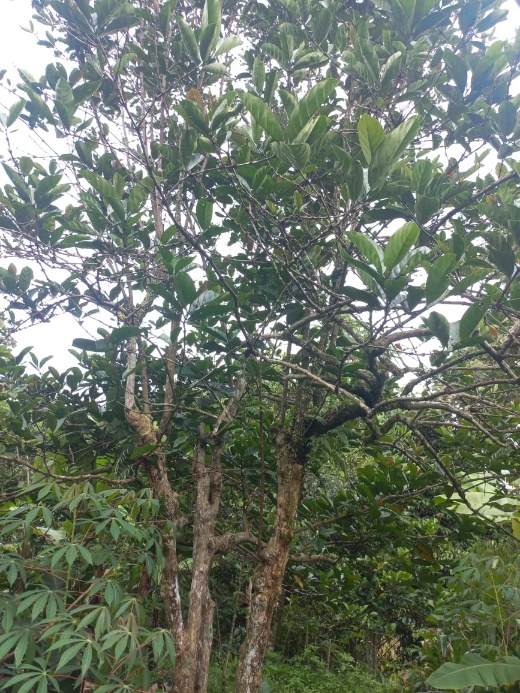 |
| SS7 | SS8 |
| 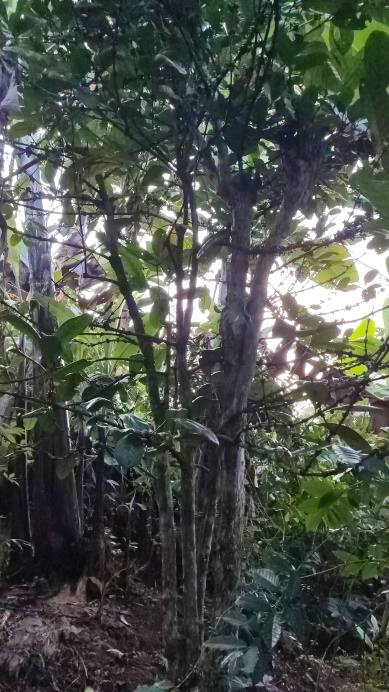 | 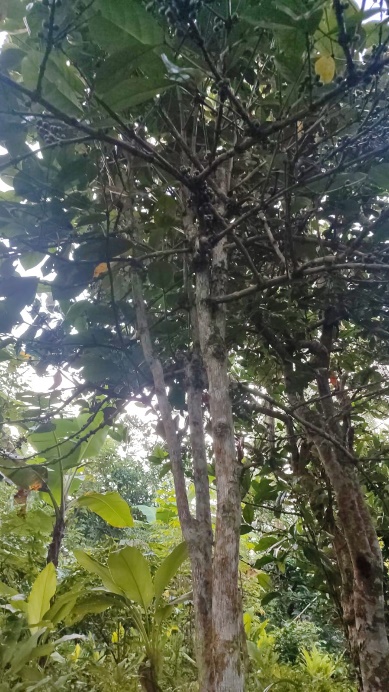 |
| SS9 | SS10 |
| 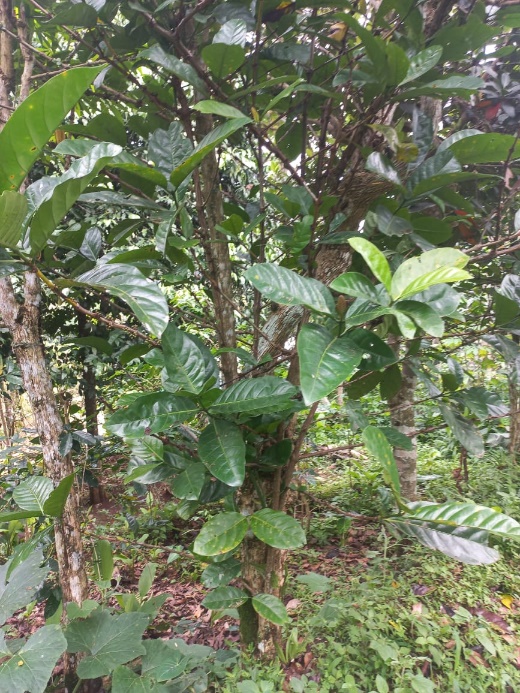 | 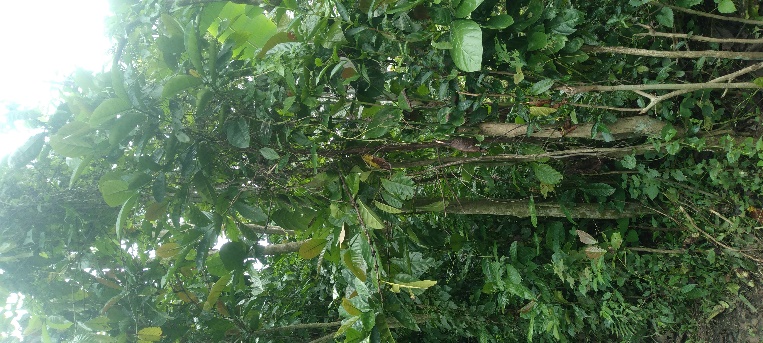 |
| SUKAWANGI, PAMULIHAN, SUMEDANG ORIGIN | |
| SP1 | SP2 |
| 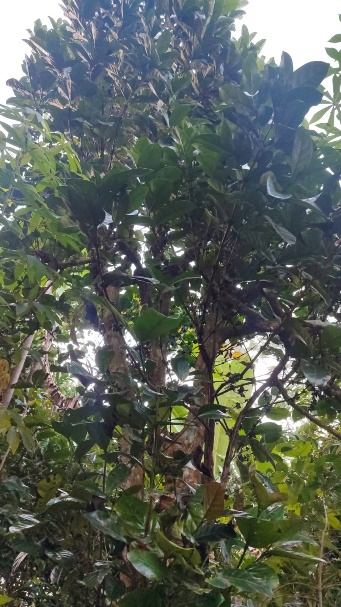 | 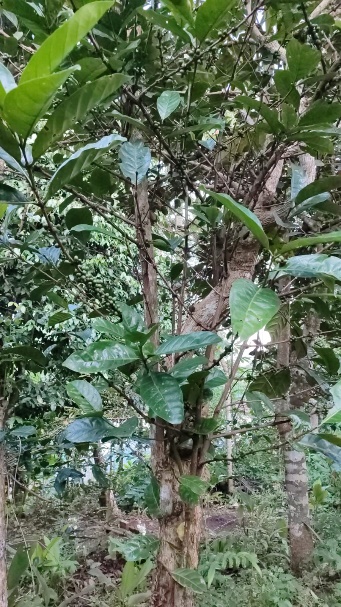 |
| SP3 | SP4 |
| 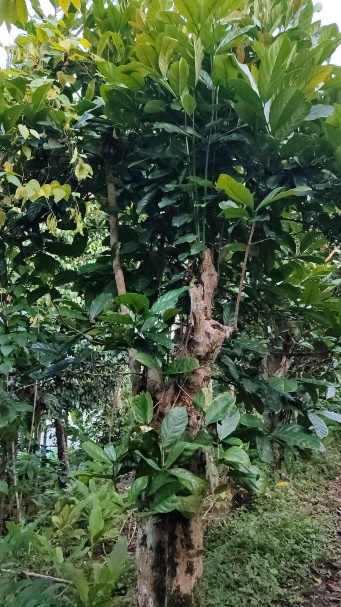 | 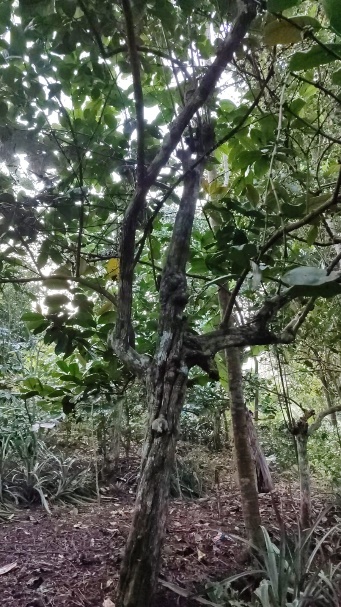 |
| SP5 | SP6 |
| 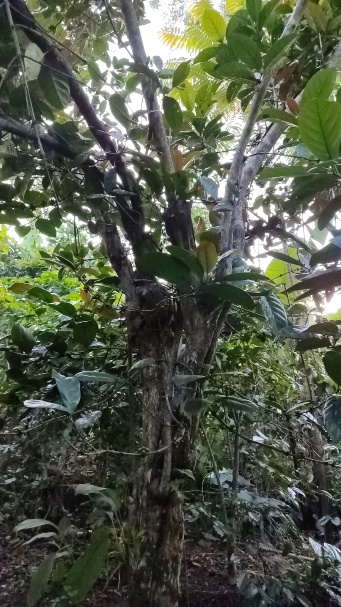 | 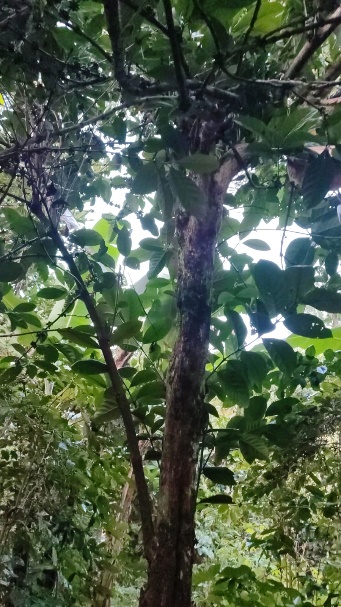 |
| SP7 | SP8 |
| 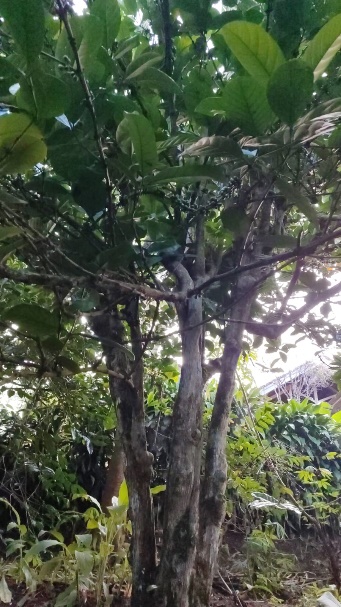 | 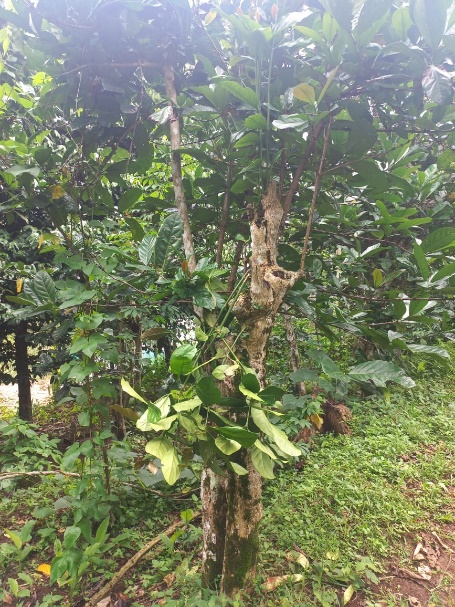 |
| SP9 | SP10 |
| 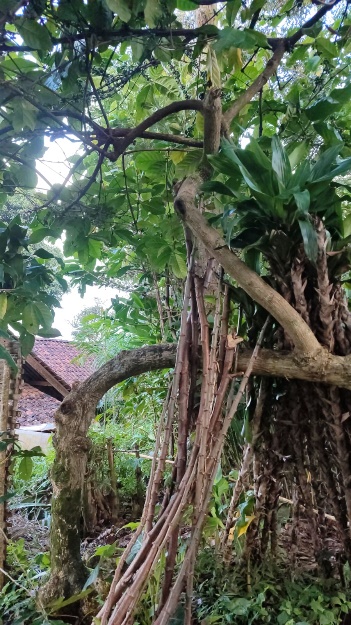 | 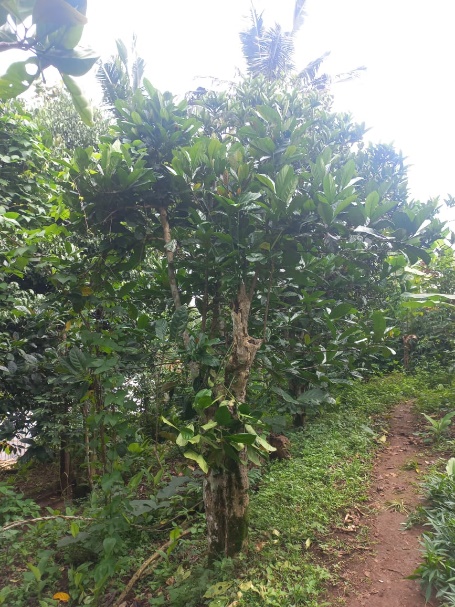 |
| SP11 | SP12 |
| 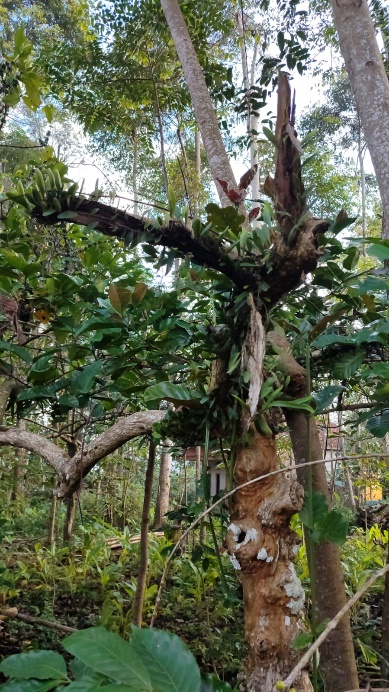 | 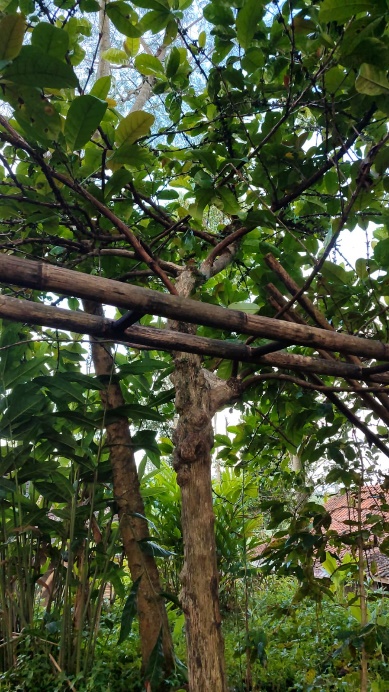 |
| SP13 | SP14 |
| 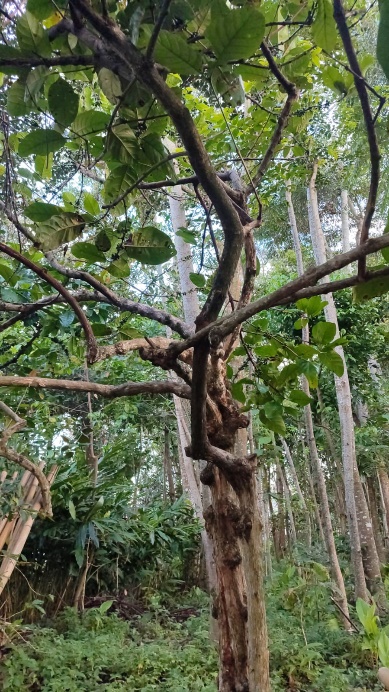 | 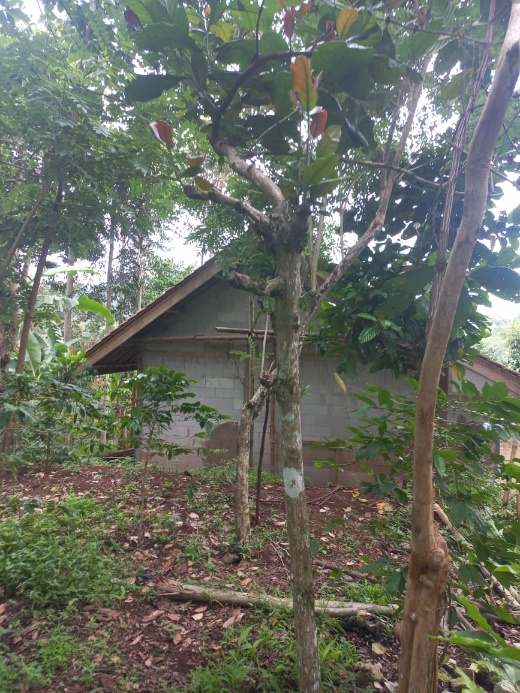 |
| SP15 | SP16 |
| 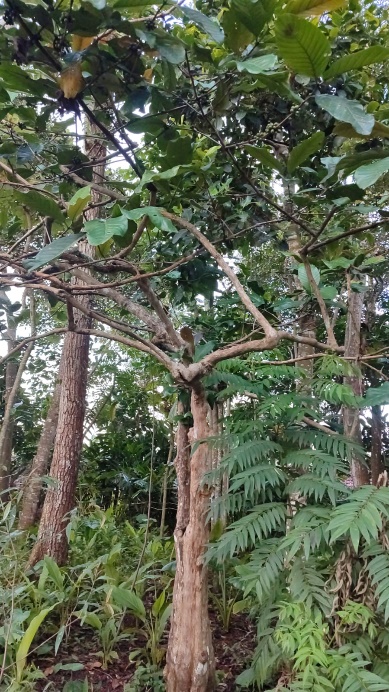 | 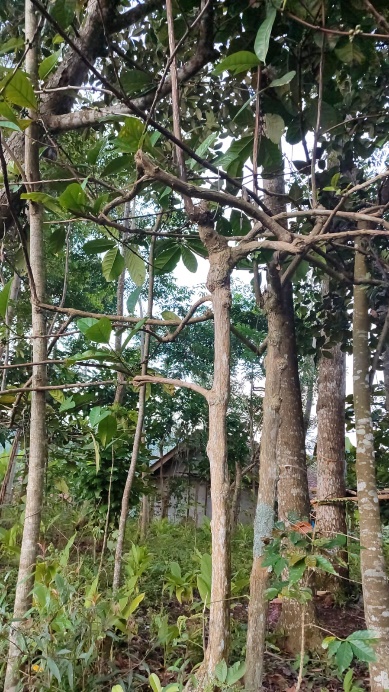 |
| SP17 | SP18 |
| 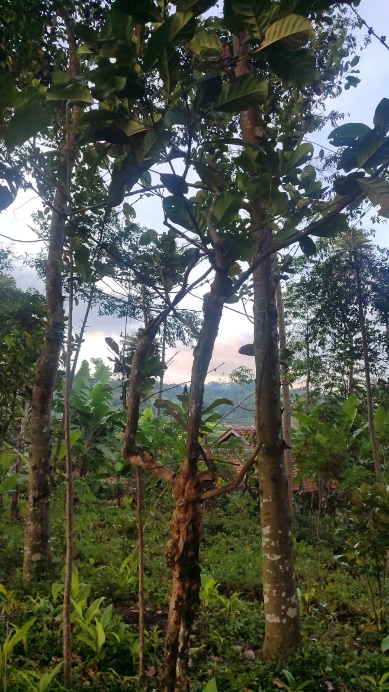 | 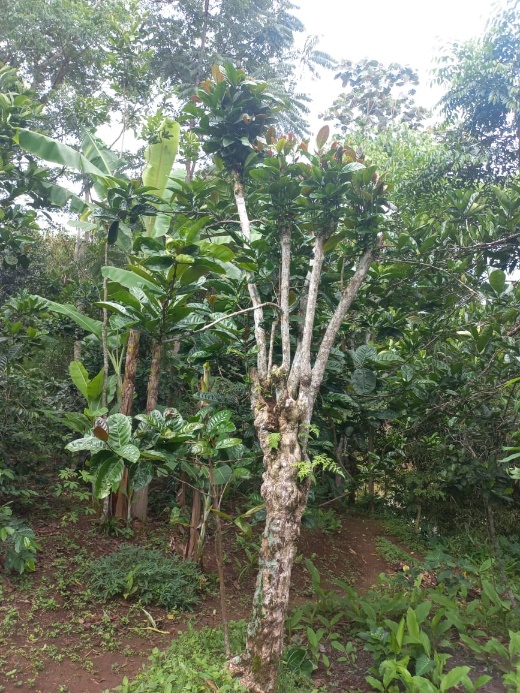 |
| SP19 | SP20 |
| 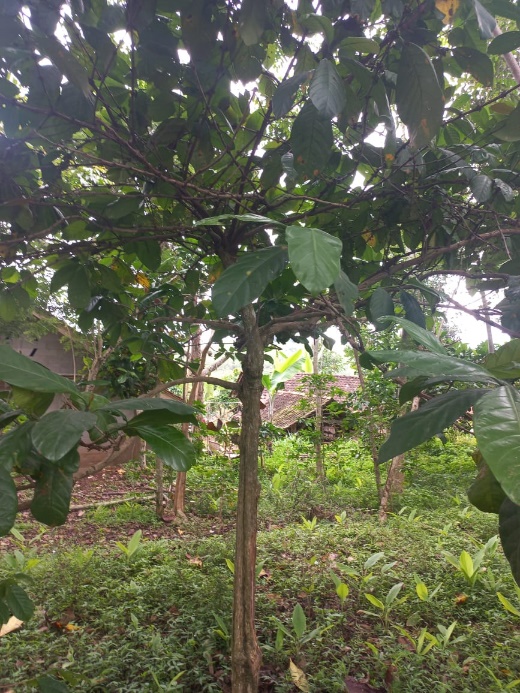 | 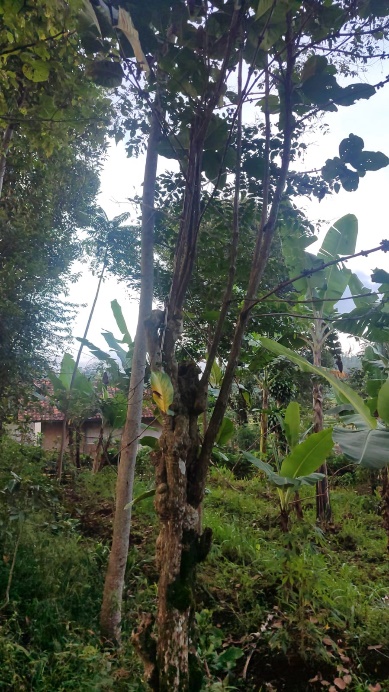 |
| TANJUNGSARI, SUMEDANG ORIGIN | |
| TS1 | TS2 |
| 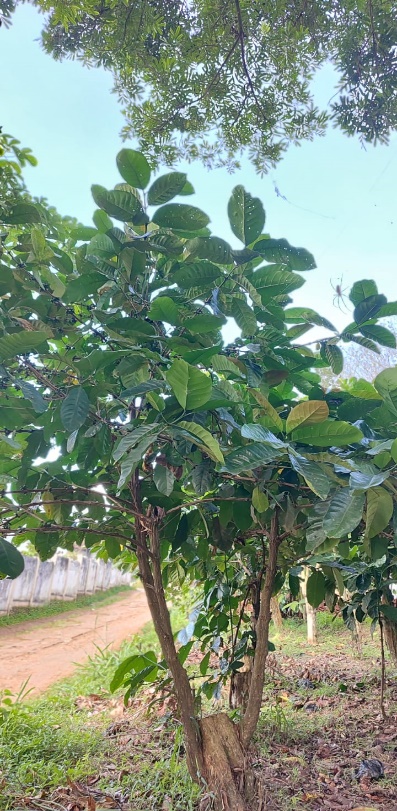 | 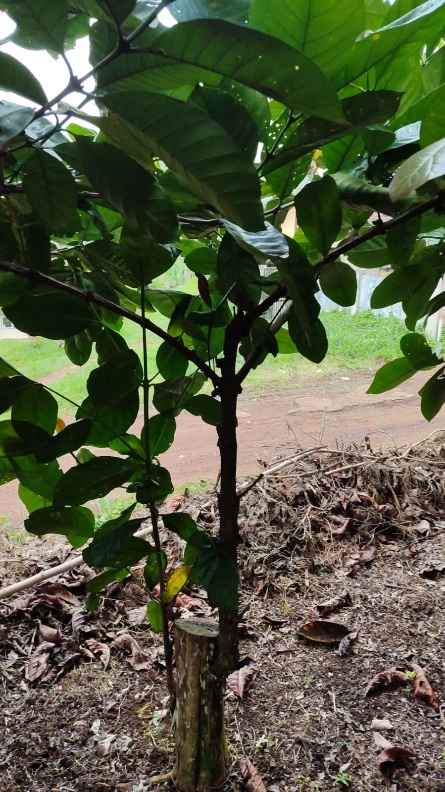 |
| TS3 | TS4 |
| 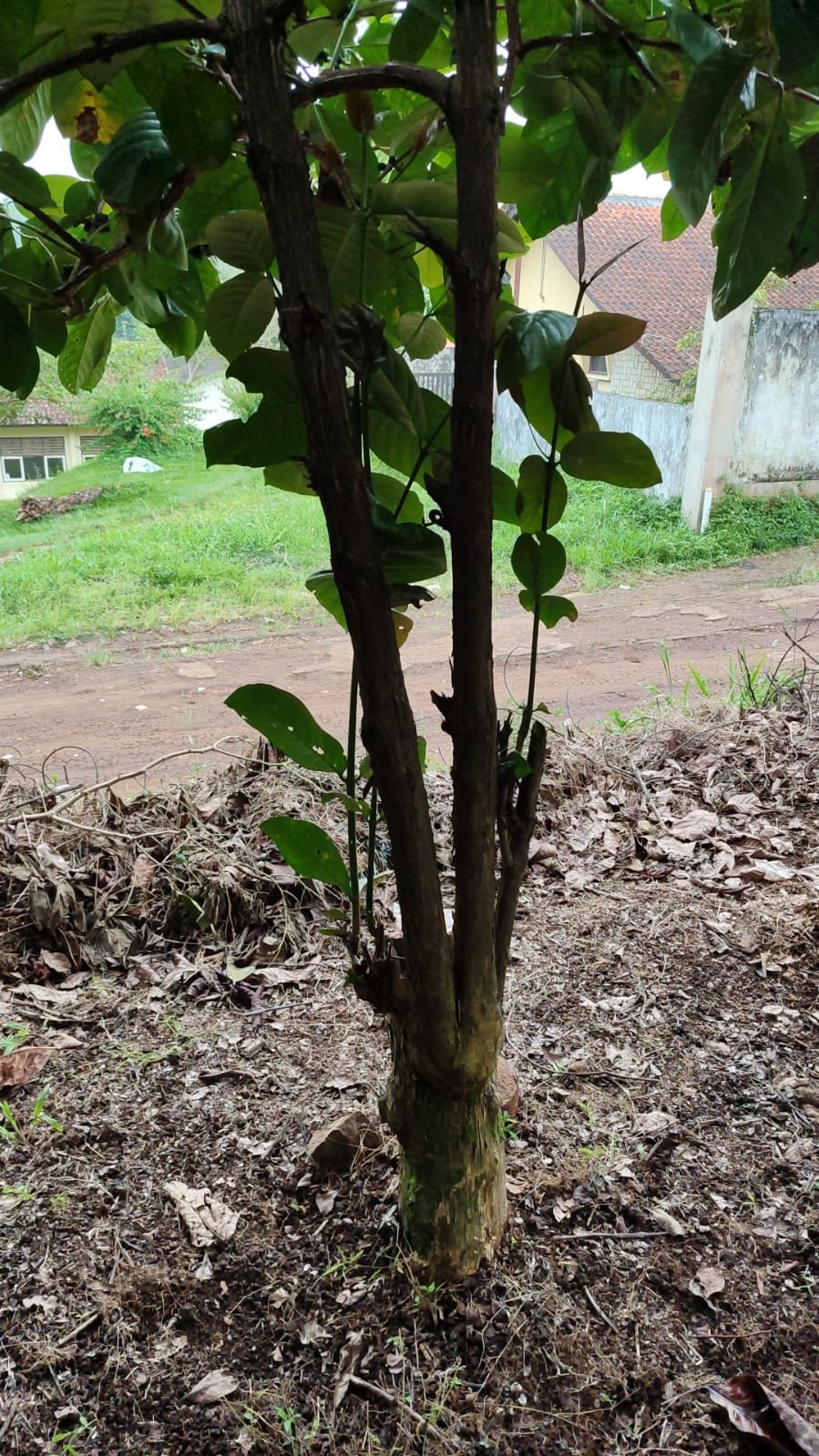 | 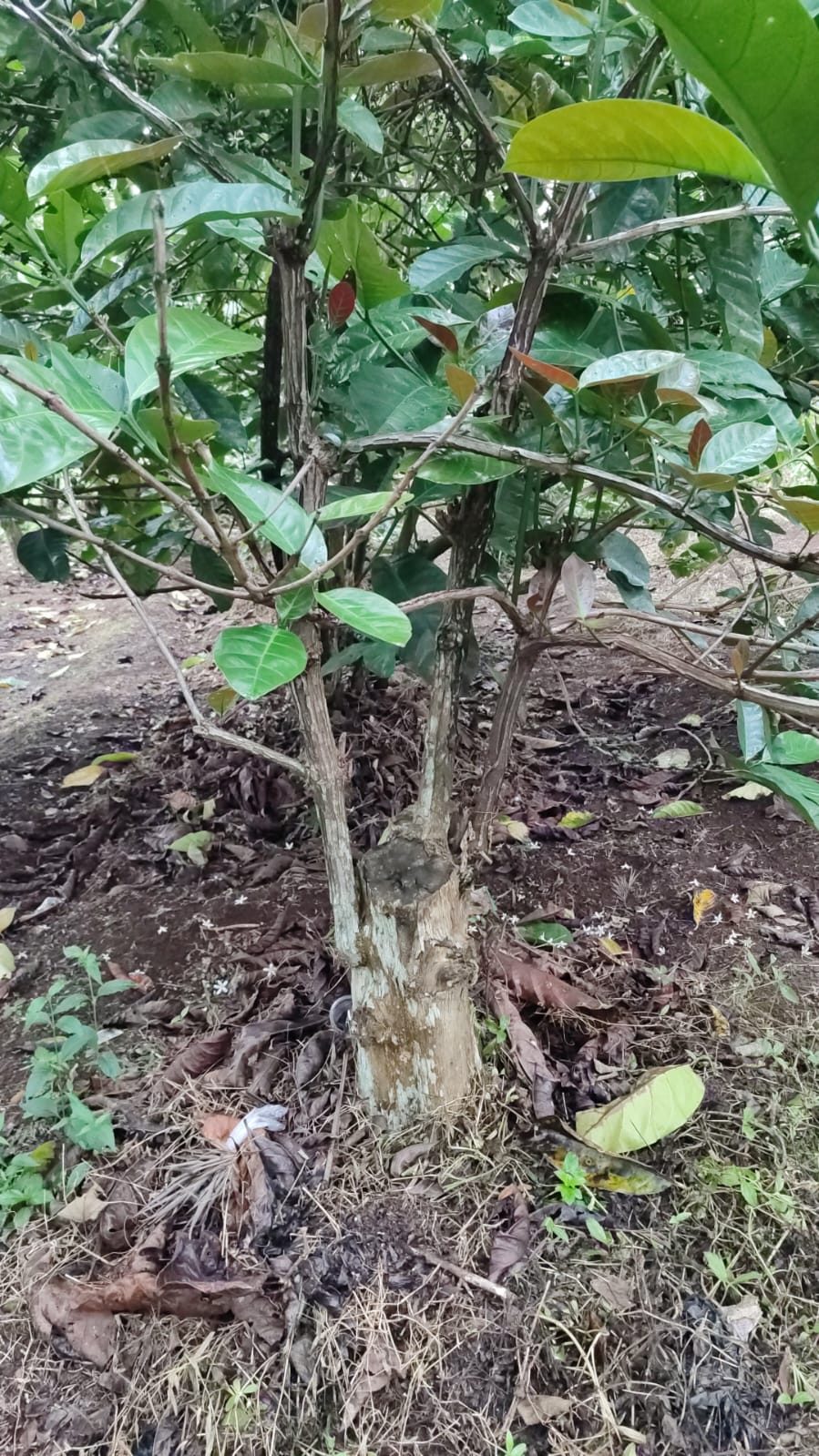 |
| TS5 | TS6 |
| 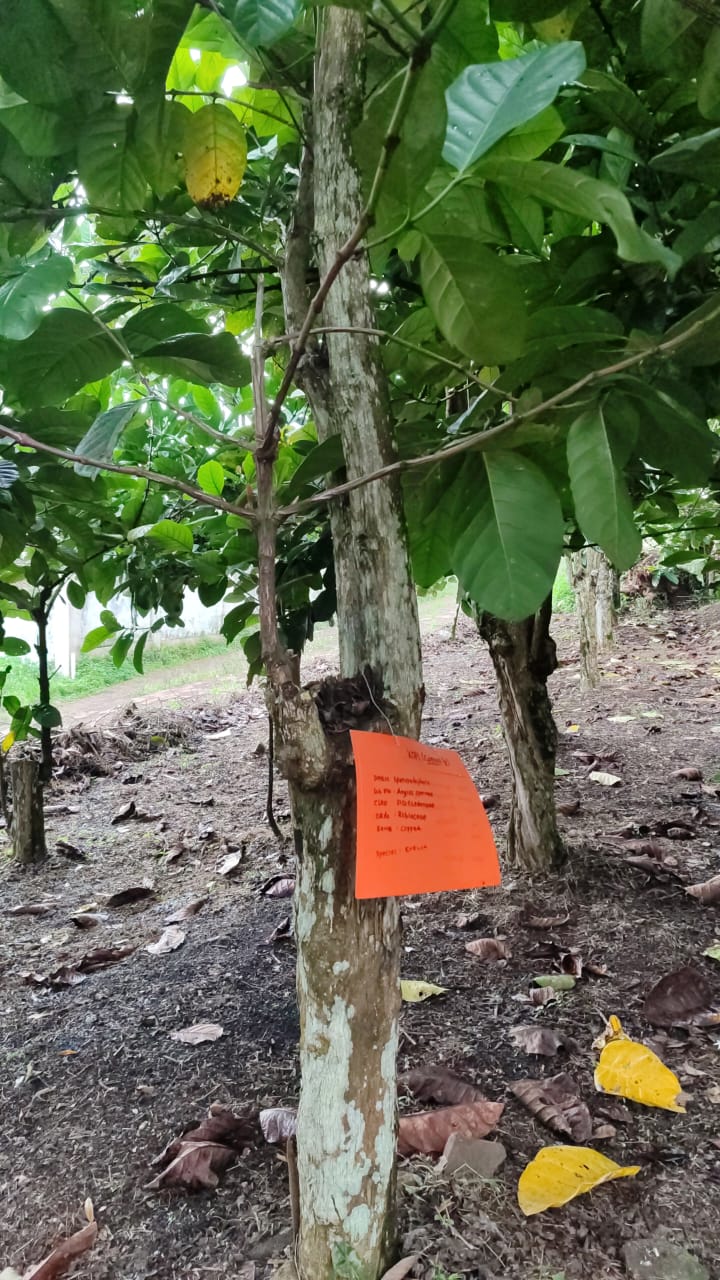 | 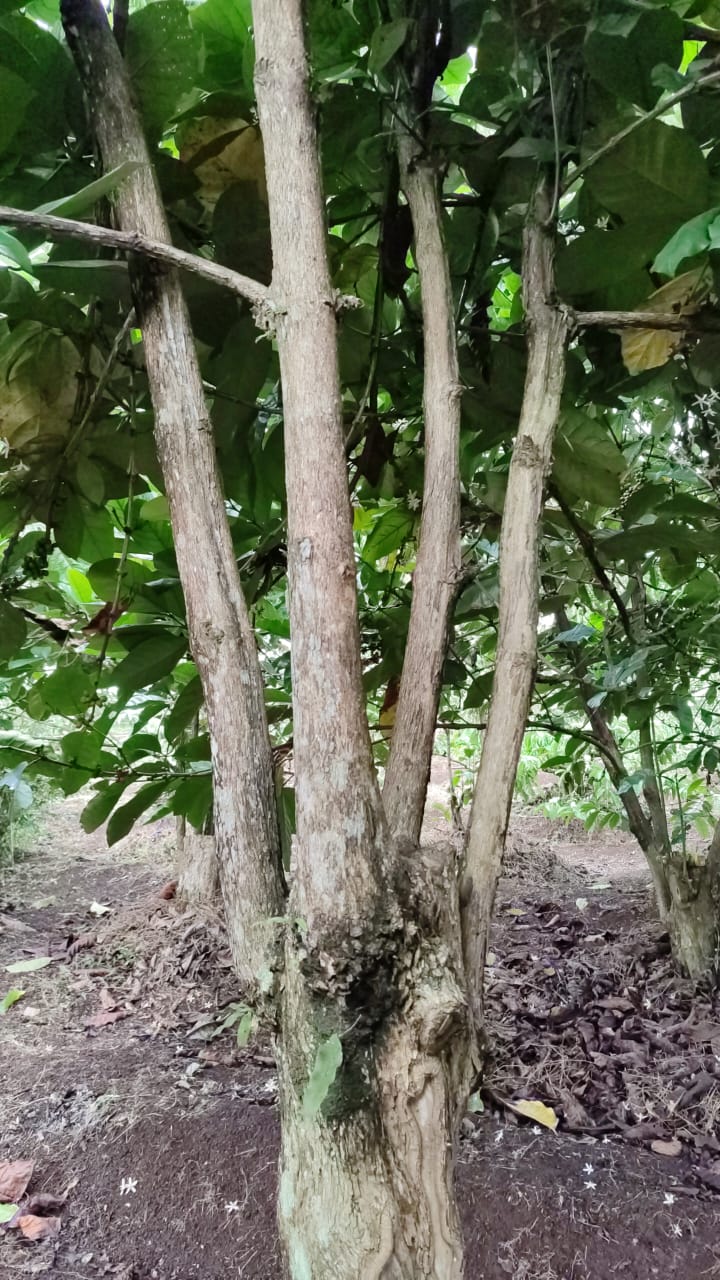 |
| TS7 |  |
| 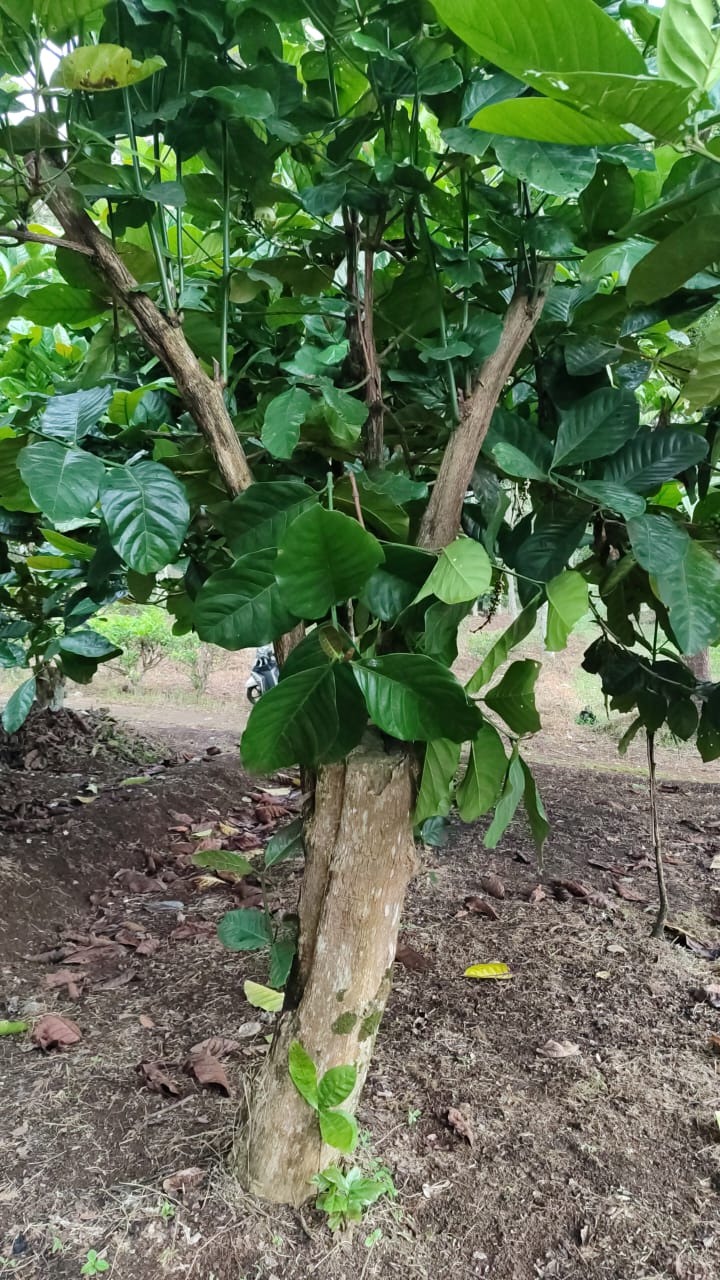 |  |
| JATINANGOR, SUMEDANG ORIGIN | |
|  | JS1 |
|  | 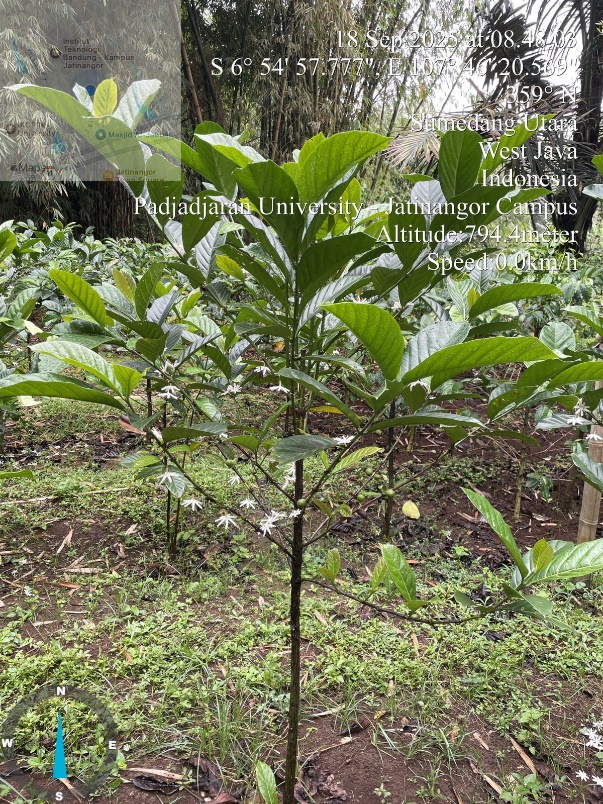 |
| JS2 | JS3 |
| 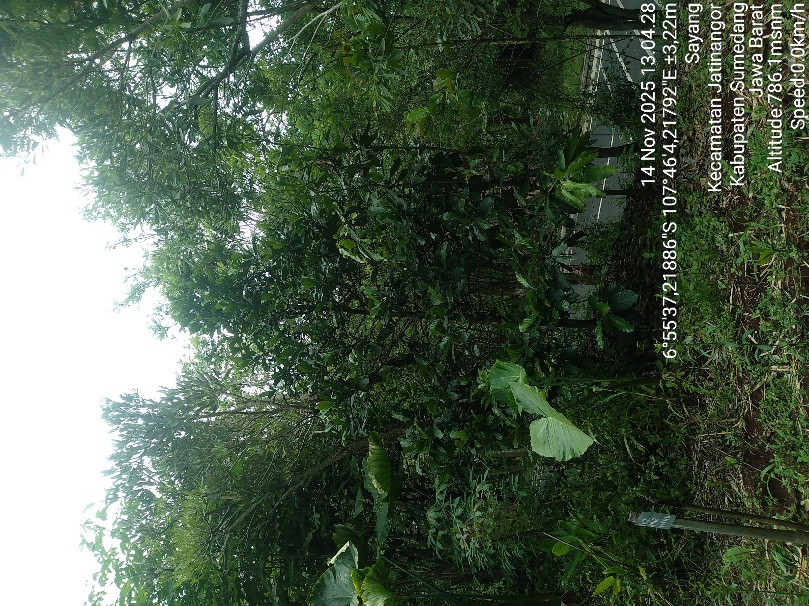 | 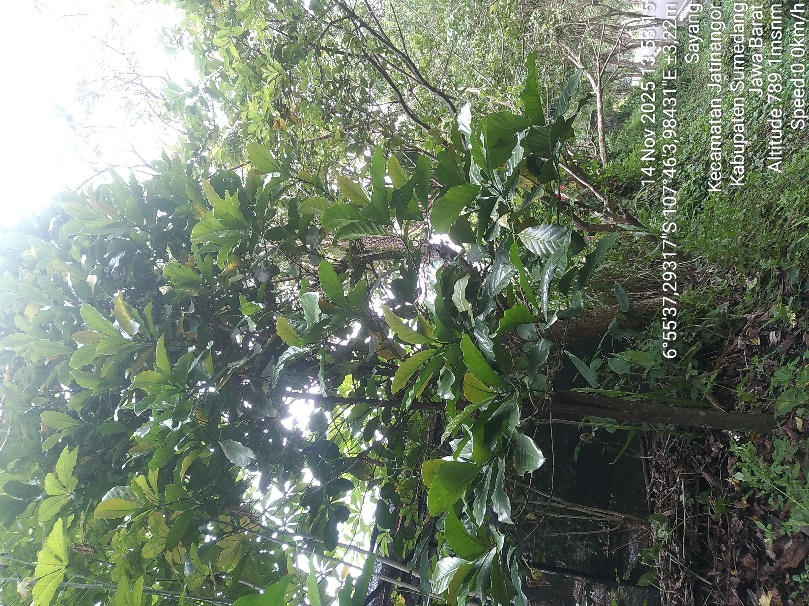 |
| MEKARBAKTI, PAMULIHAN, SUMEDANG ORIGIN | |
| MB1 | MB2 |
| 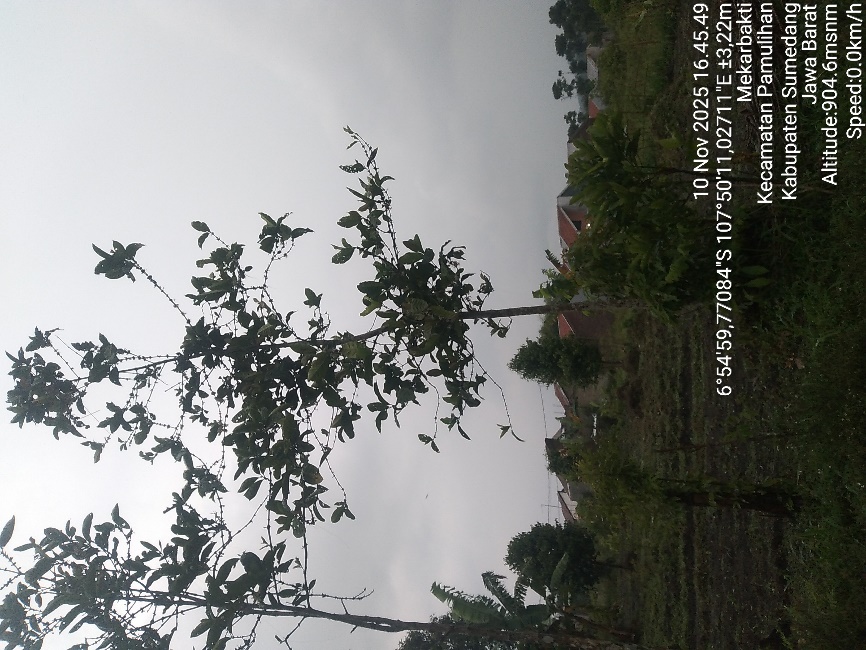 | 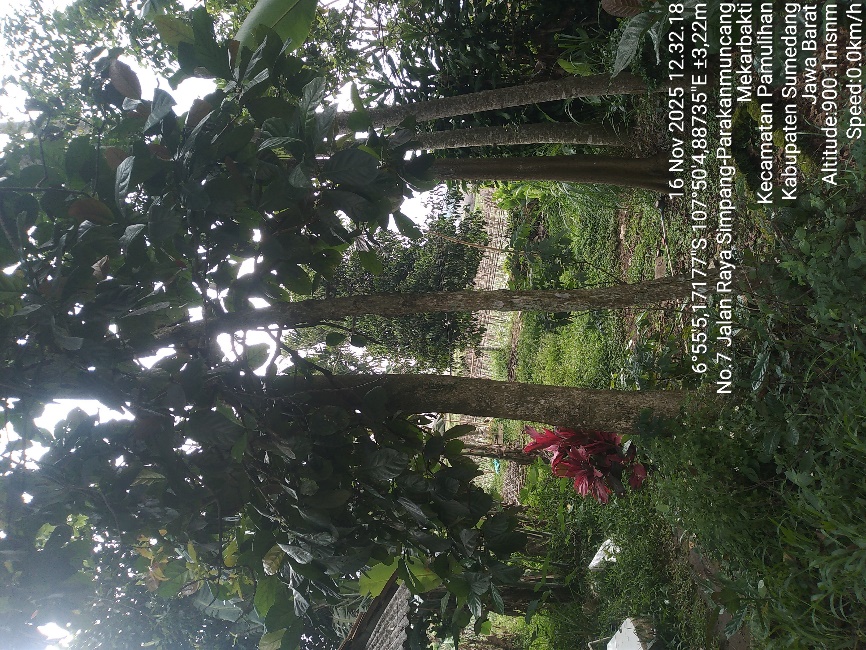 |
| MB3 | MB4 |
| 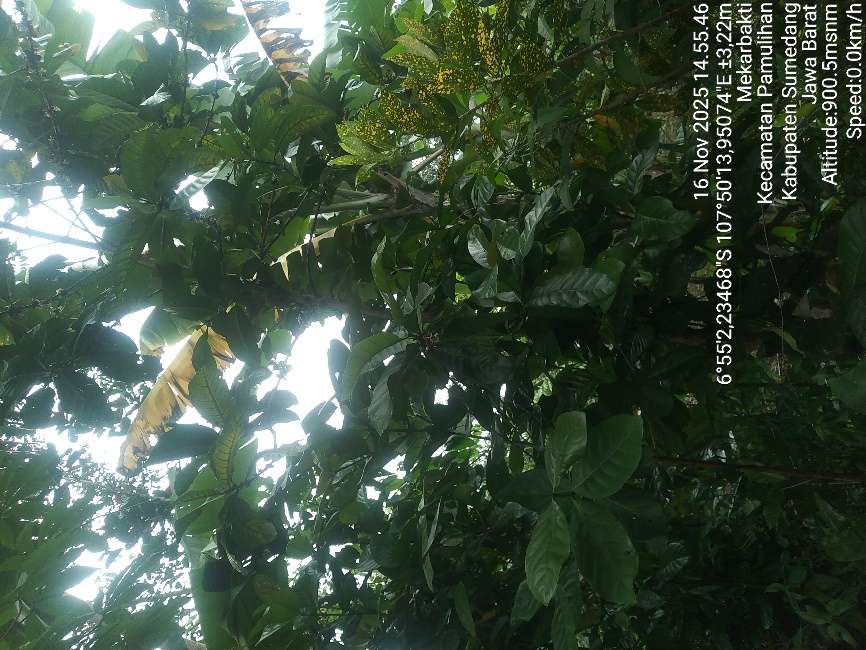 | 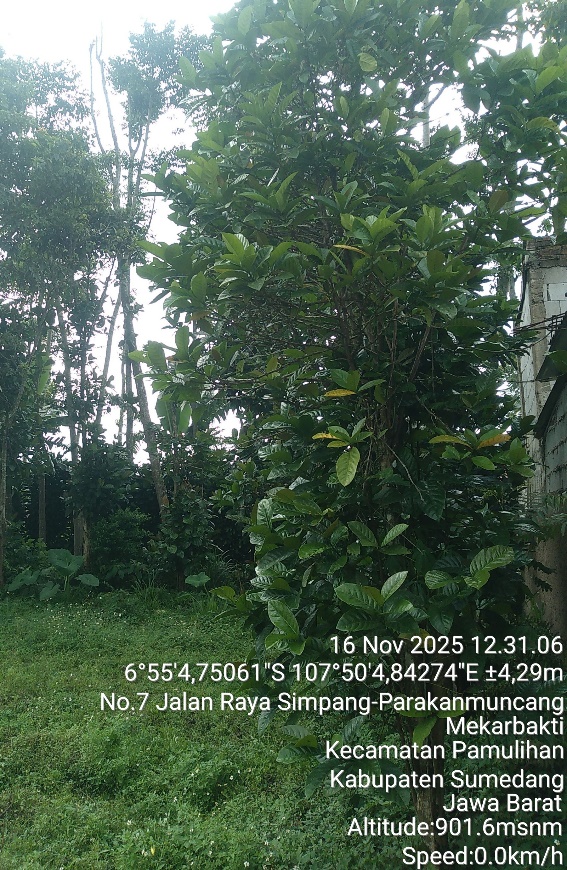 |
| SUKABUMI ORIGIN | |
| LB1 | LB2 |
| 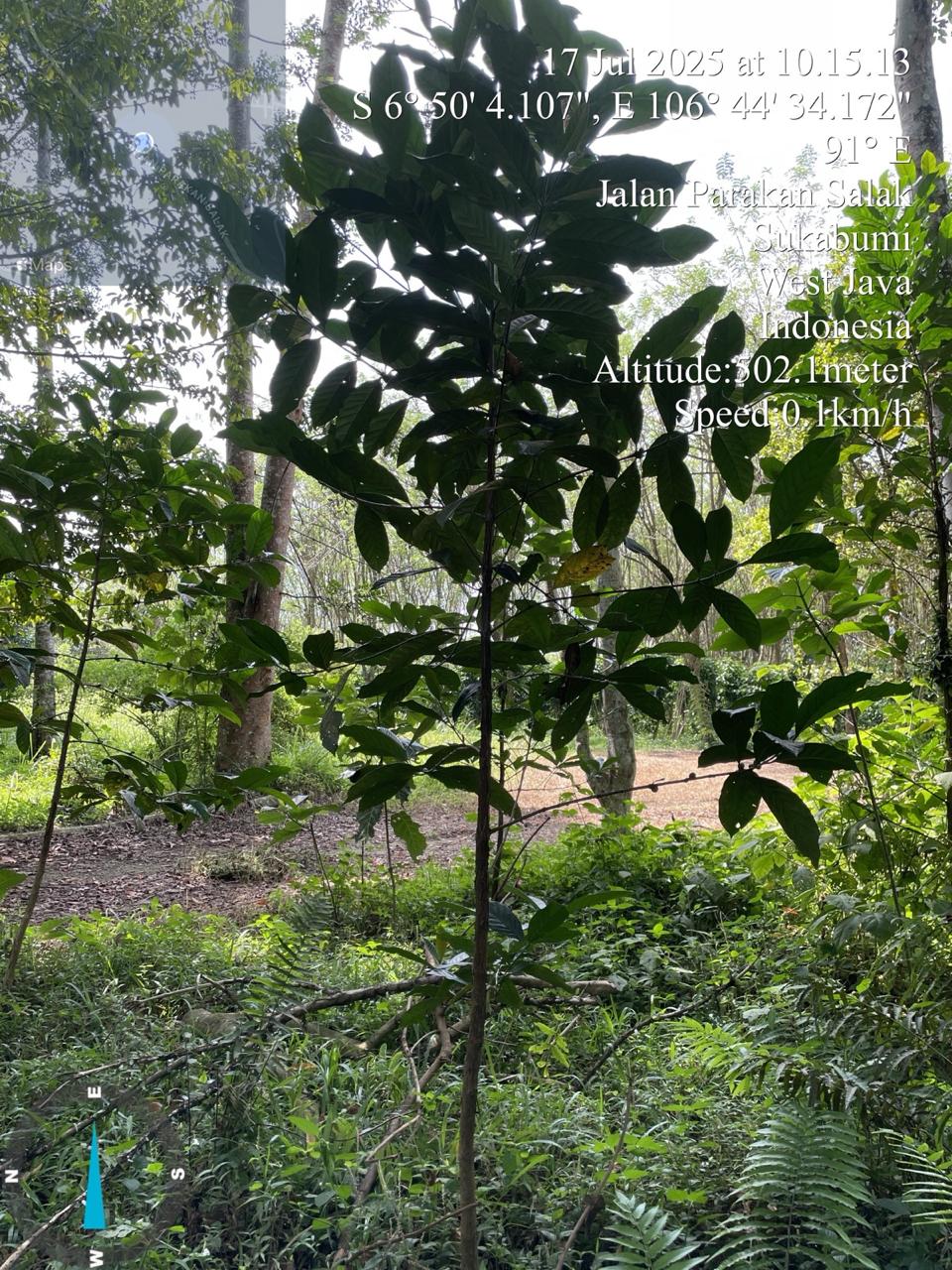 | 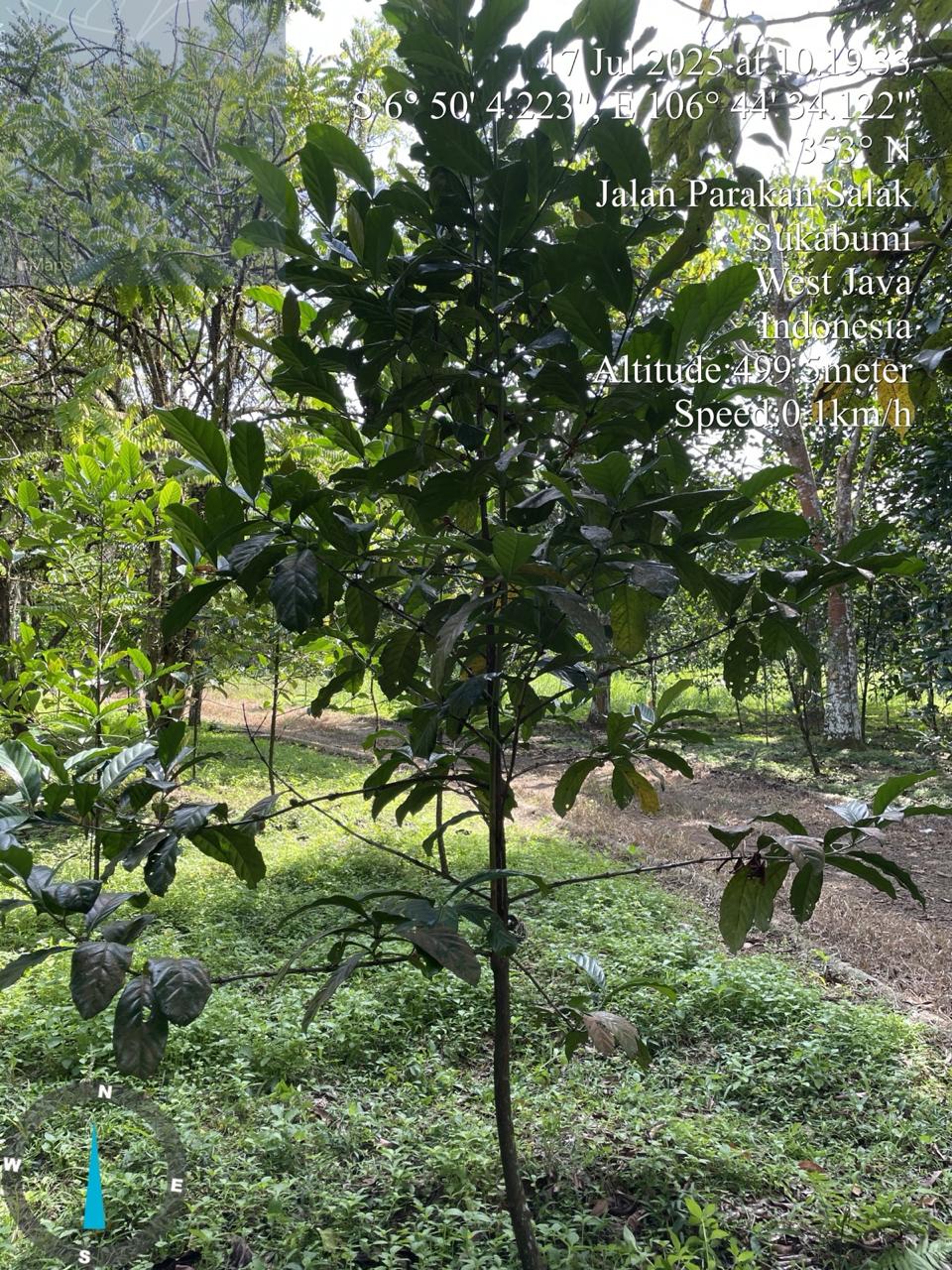 |
| LB3 | LB4 |
| 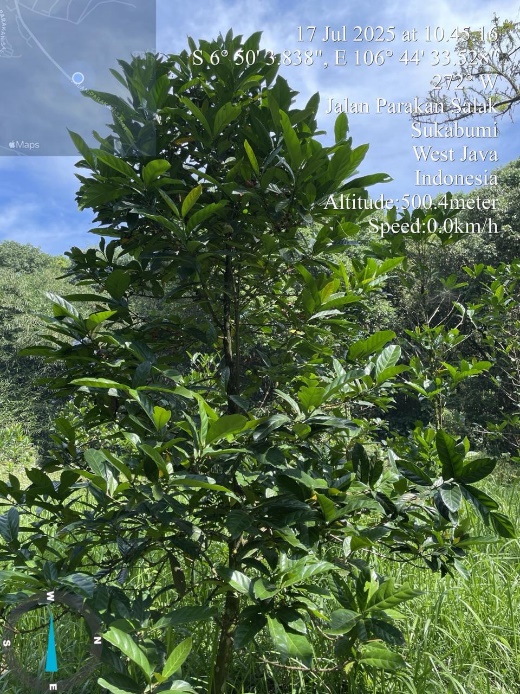 | 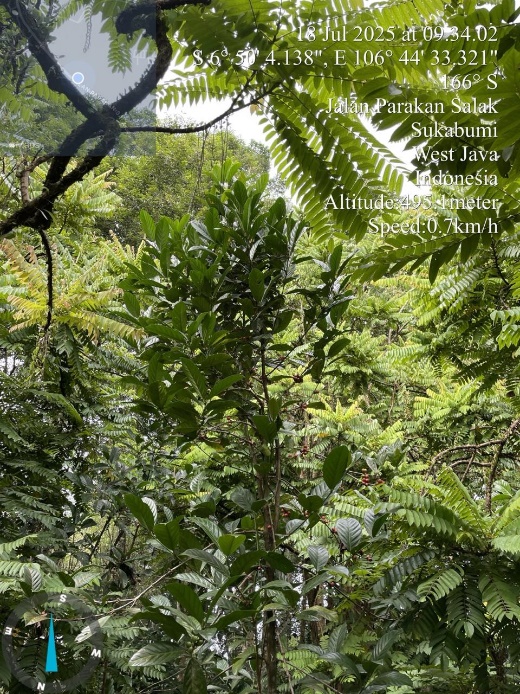 |
| LB5 | LB6 |
| 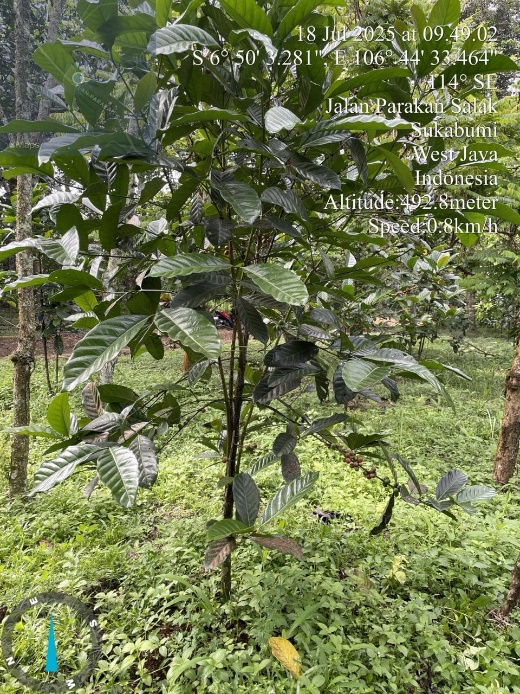 | 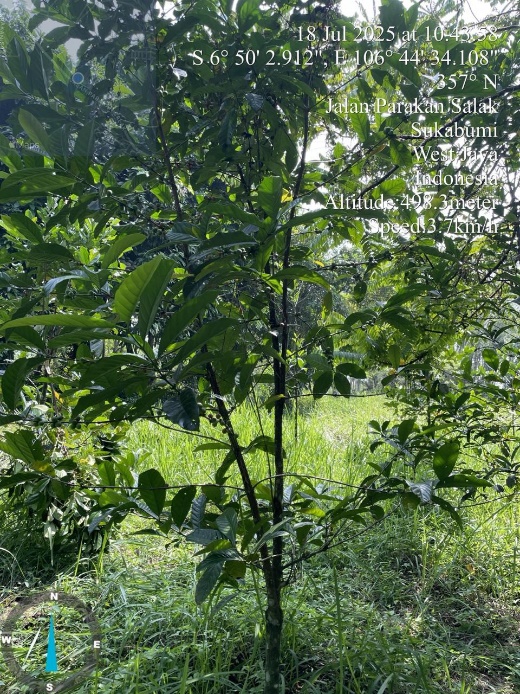 |
| LB7 | LB8 |
| 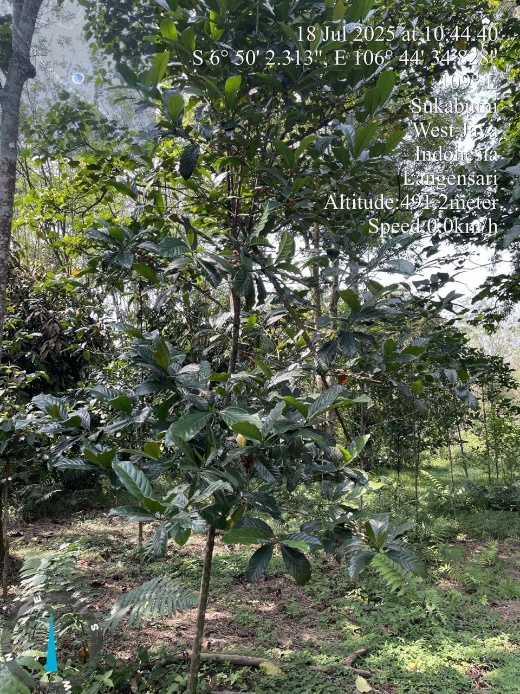 | 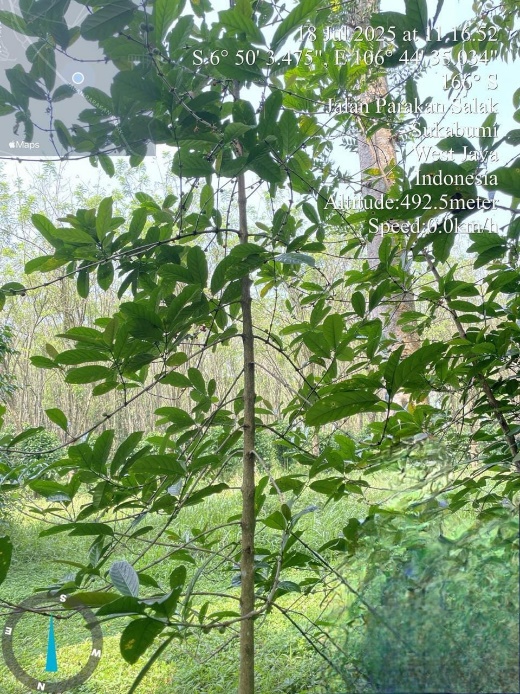 |
| LB9 | LB10 |
| 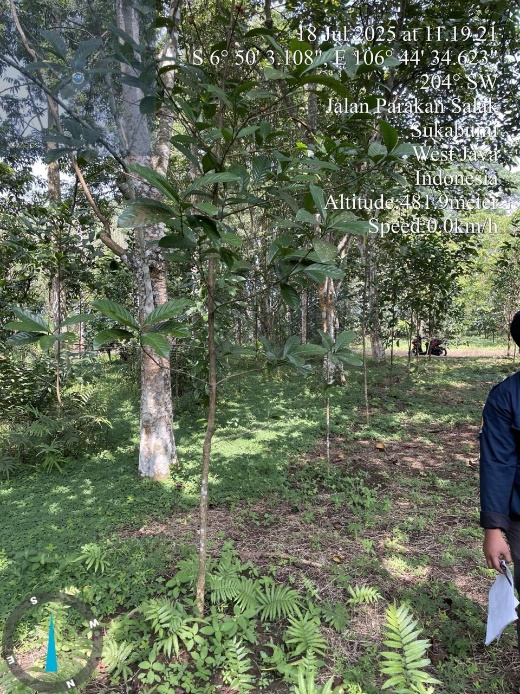 | 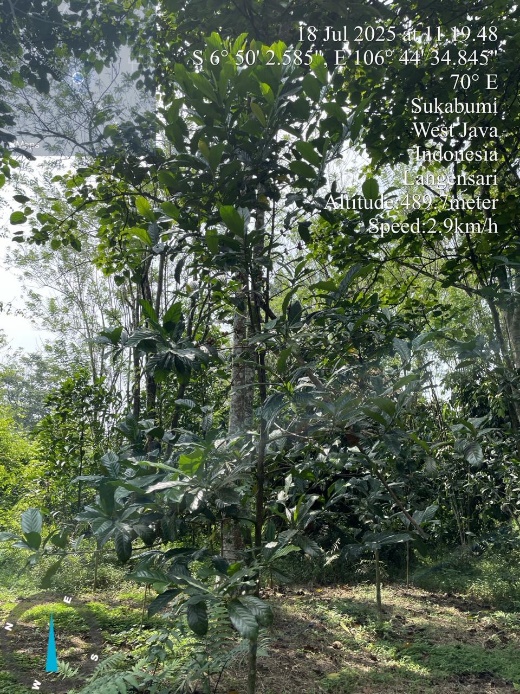 |
| LB11 | EB1 |
| 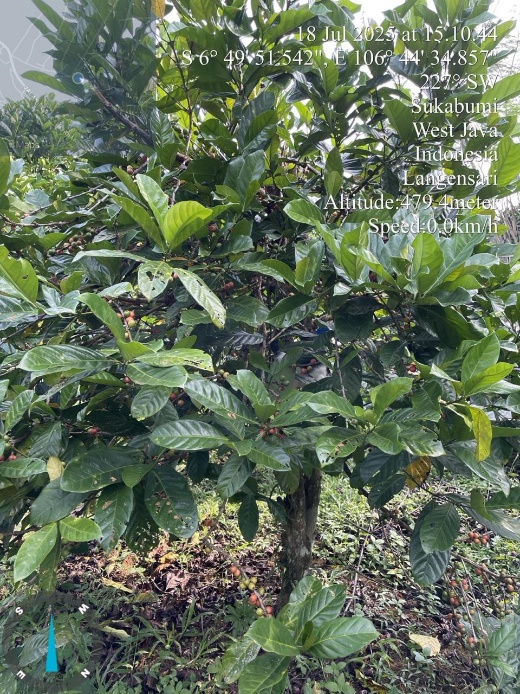 | 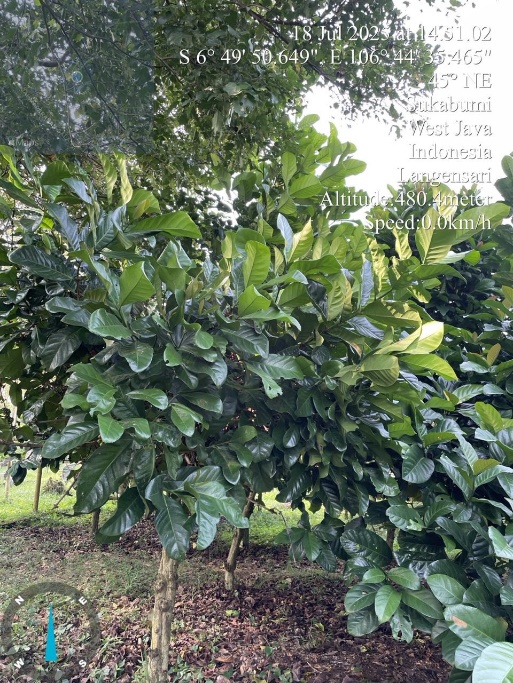 |
| EB2 | JMP1 |
| 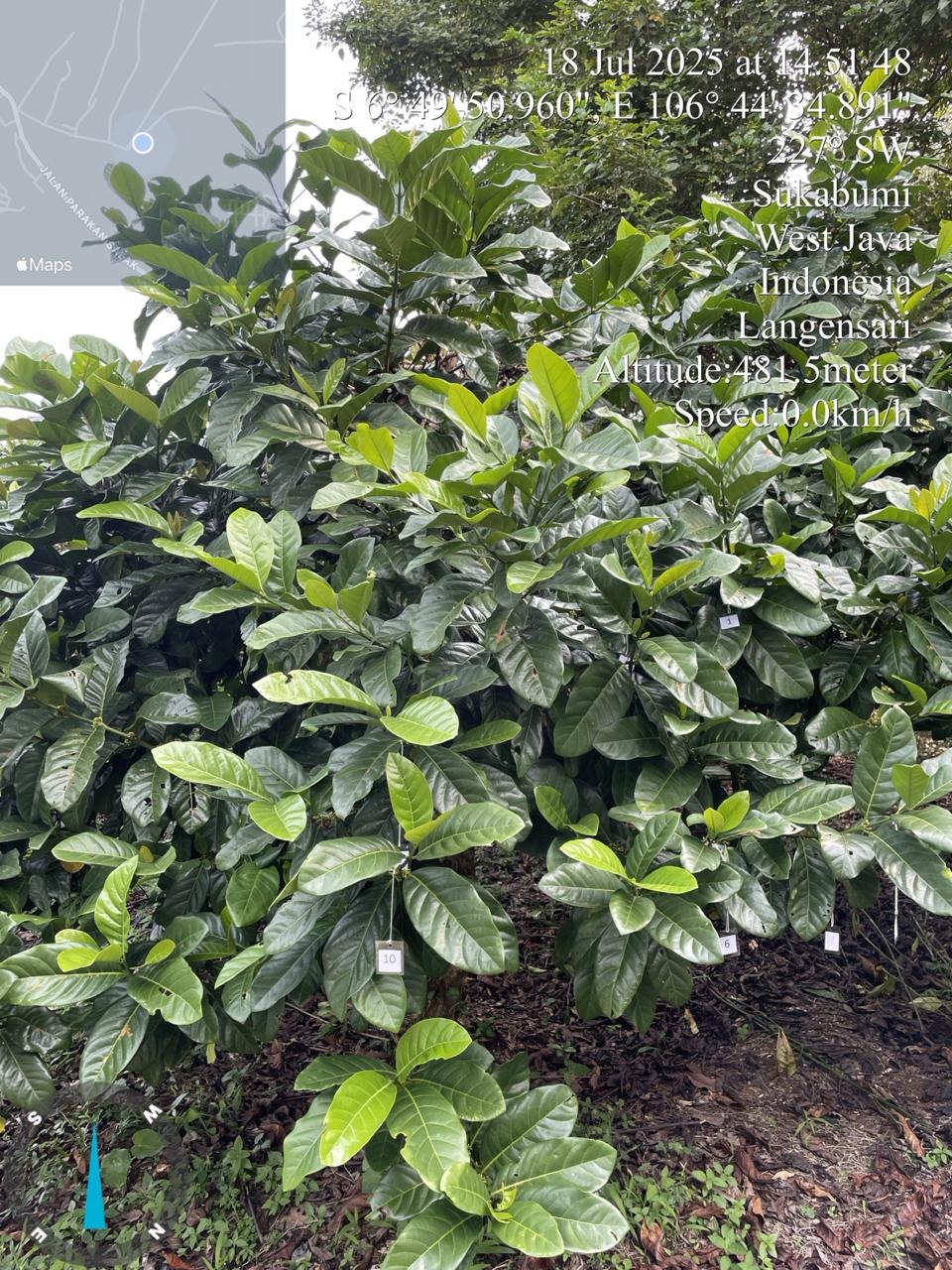 | 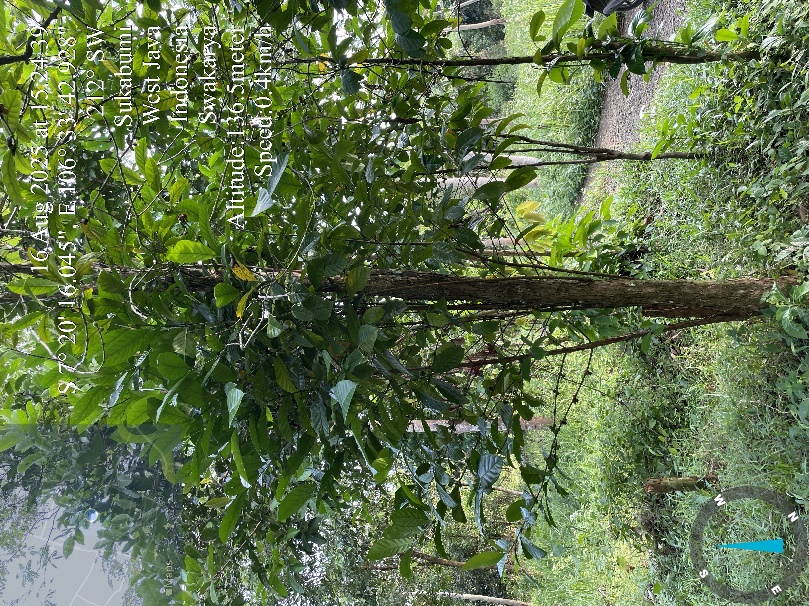 |
| JMP2 | JMP3 |
| 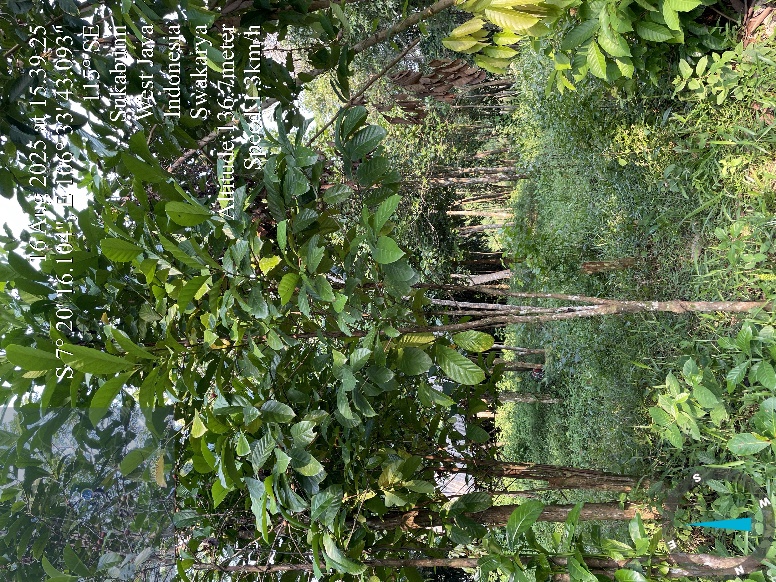 | 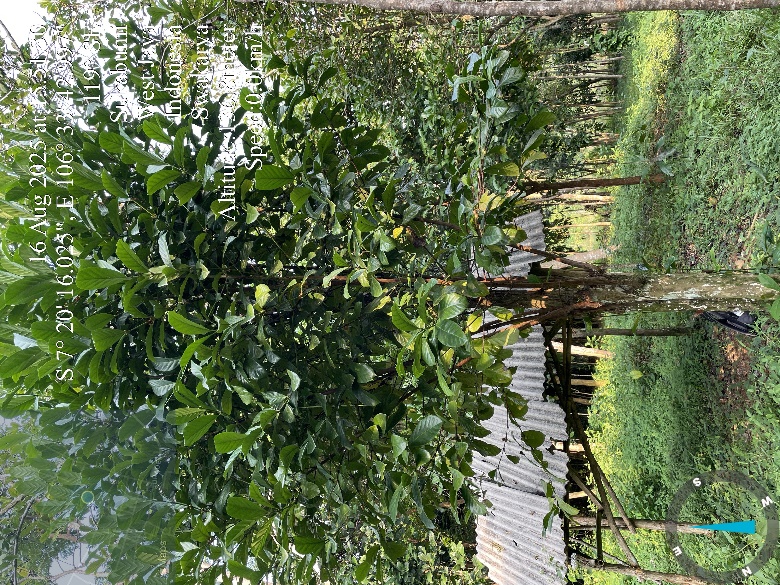 |
| JMP4 | JMP5 |
| 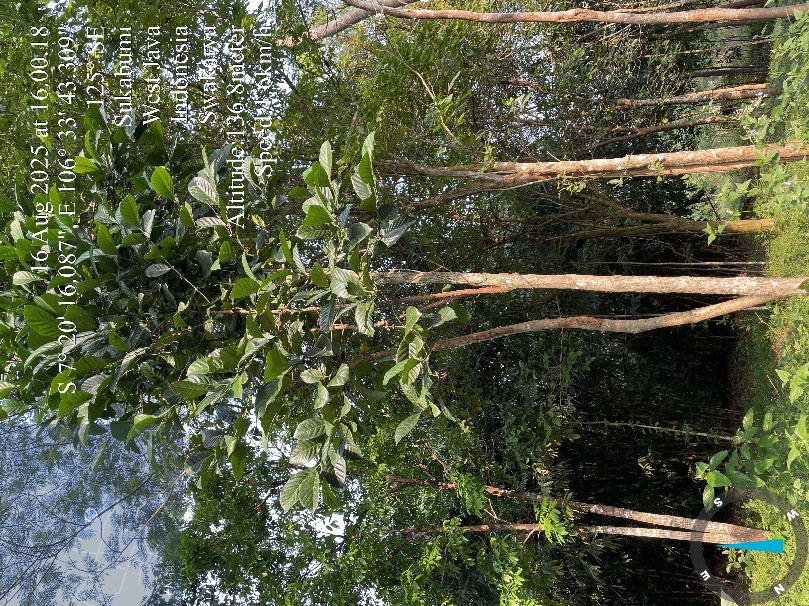 | 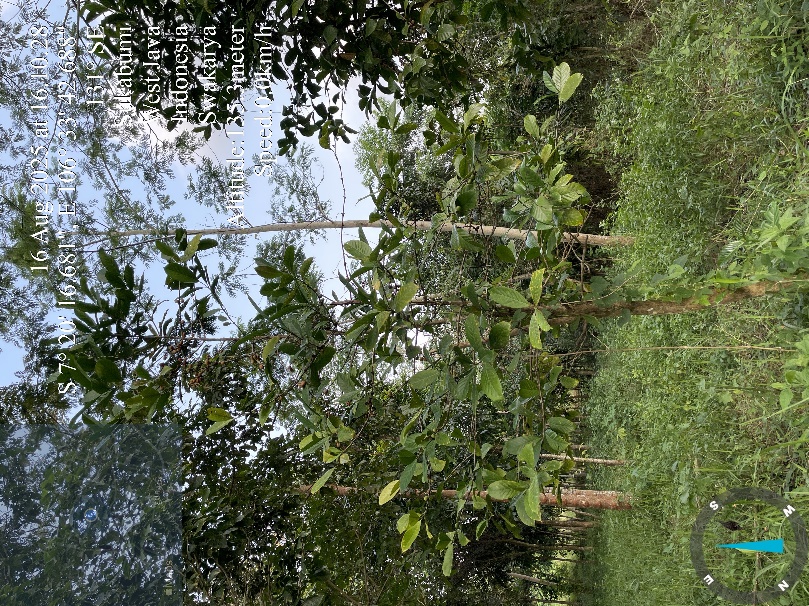 |
| JMP6 | JMP7 |
| 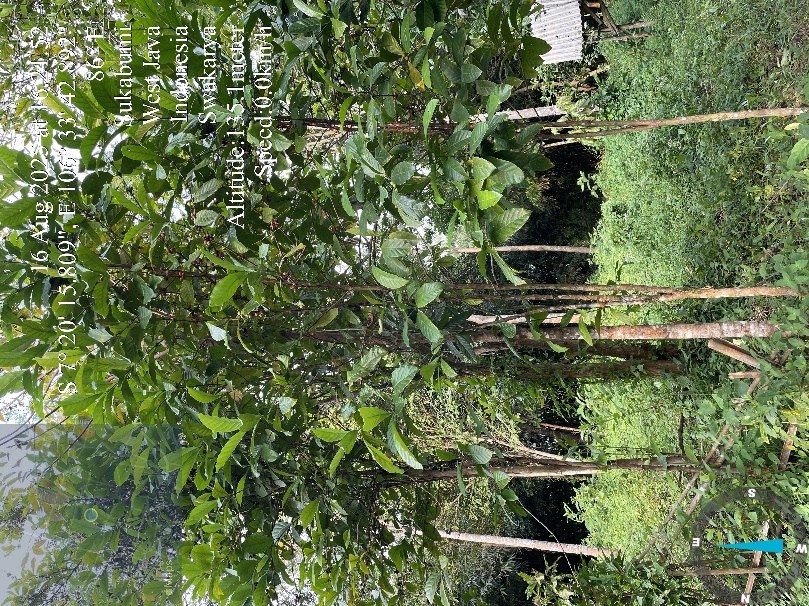 | 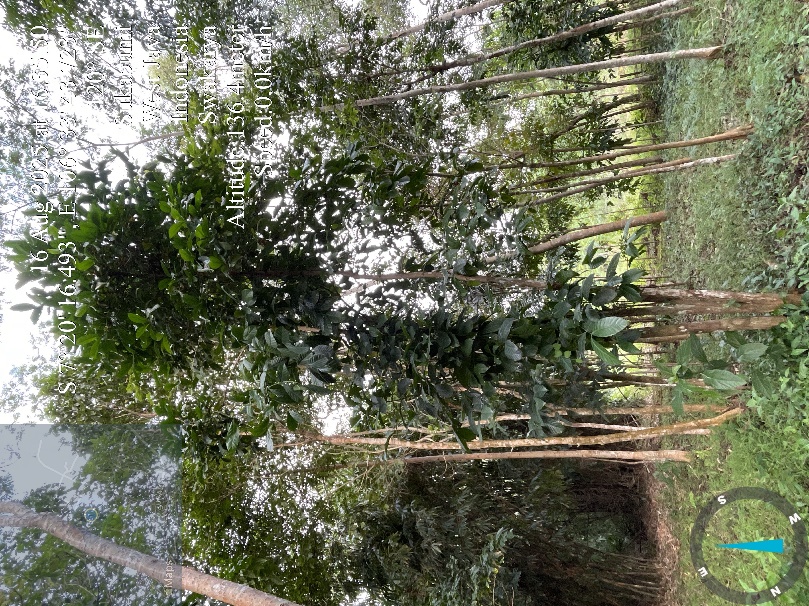 |
| CIPASUNG, DARMA, KUNINGAN ORIGIN | |
| CDK1 | CDK2 |
| 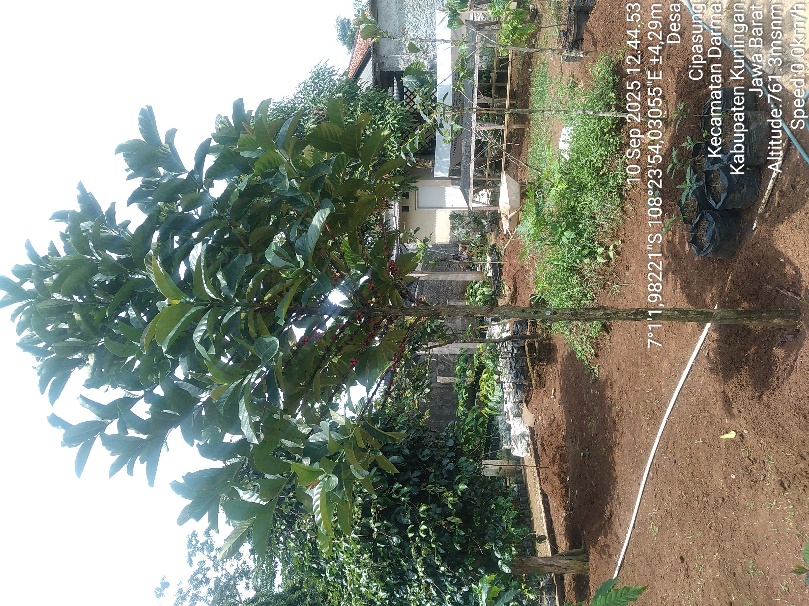 | 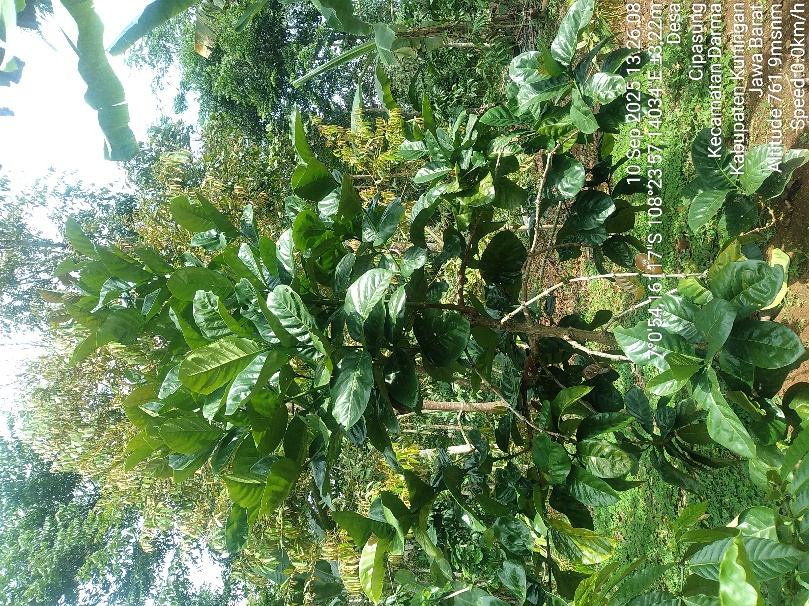 |
| CDK3 | CDK4 |
| 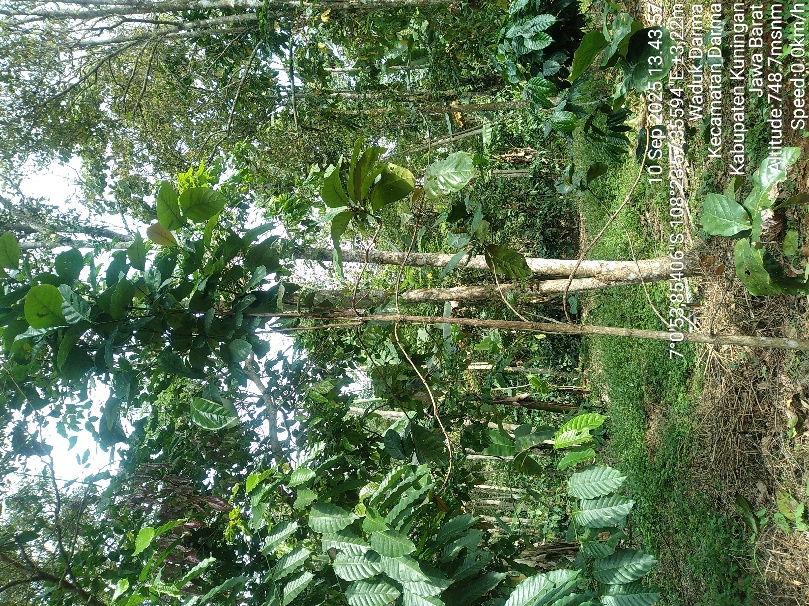 | 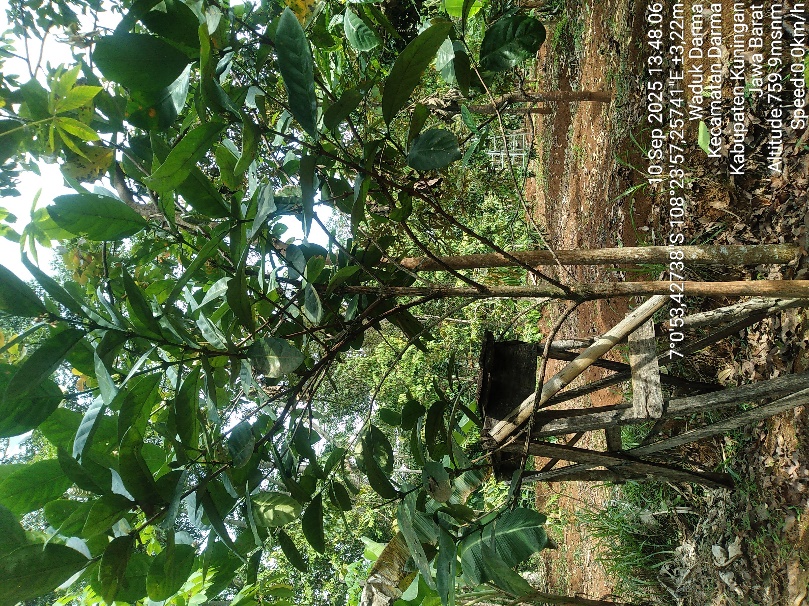 |
| CDK5 | CDK6 |
| 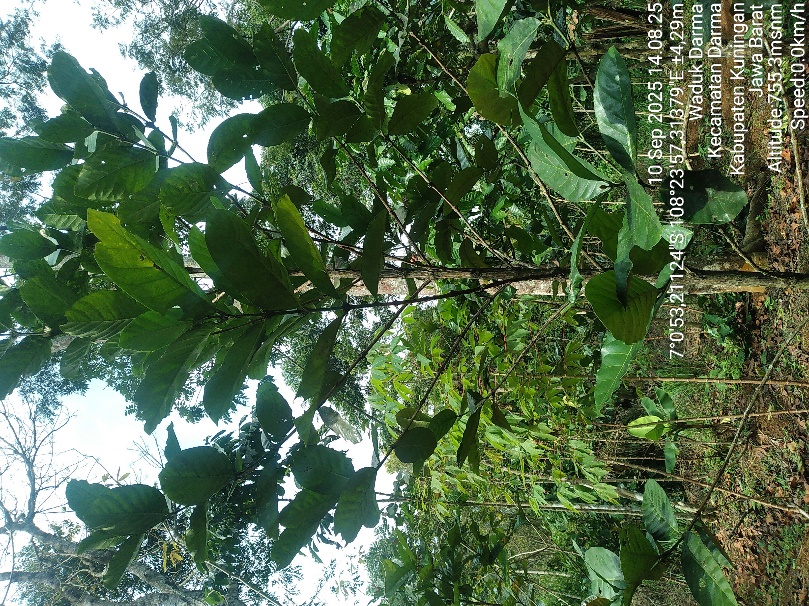 | 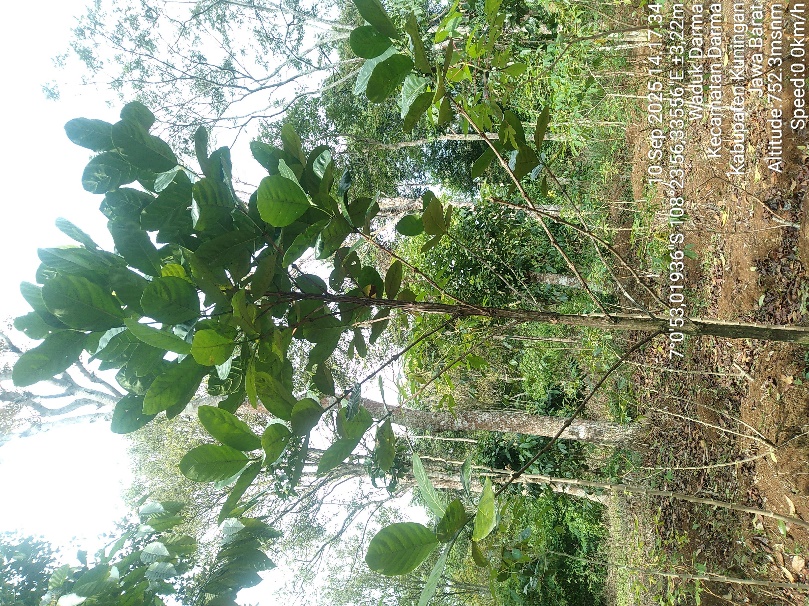 |
| CDK7 | CDK8 |
| 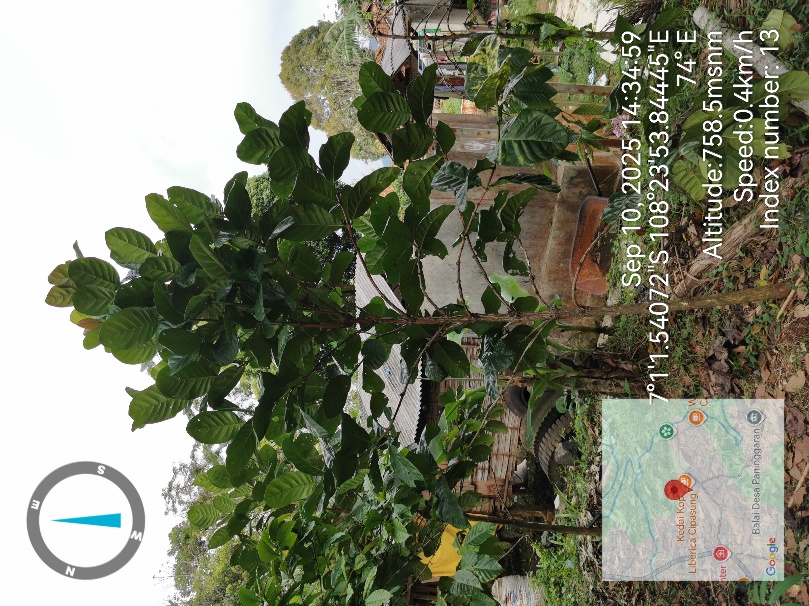 | 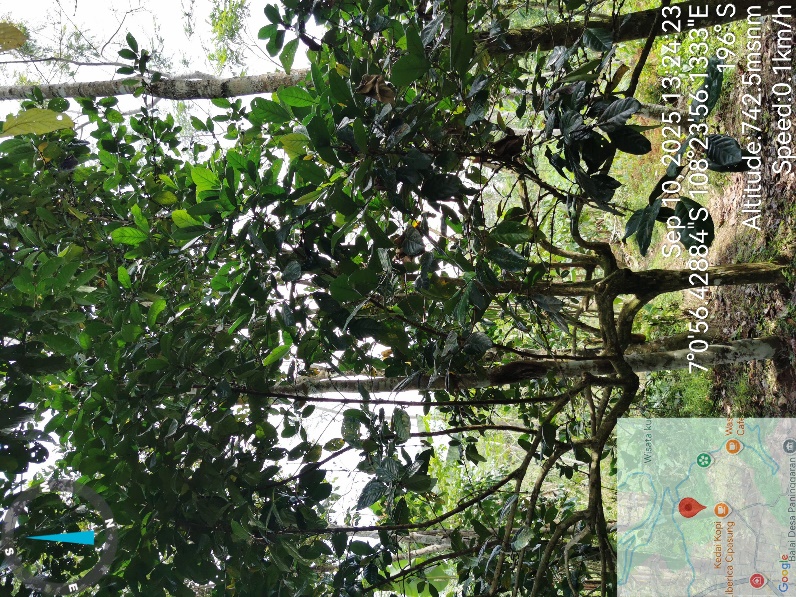 |
| CDK9 | CDK10 |
| 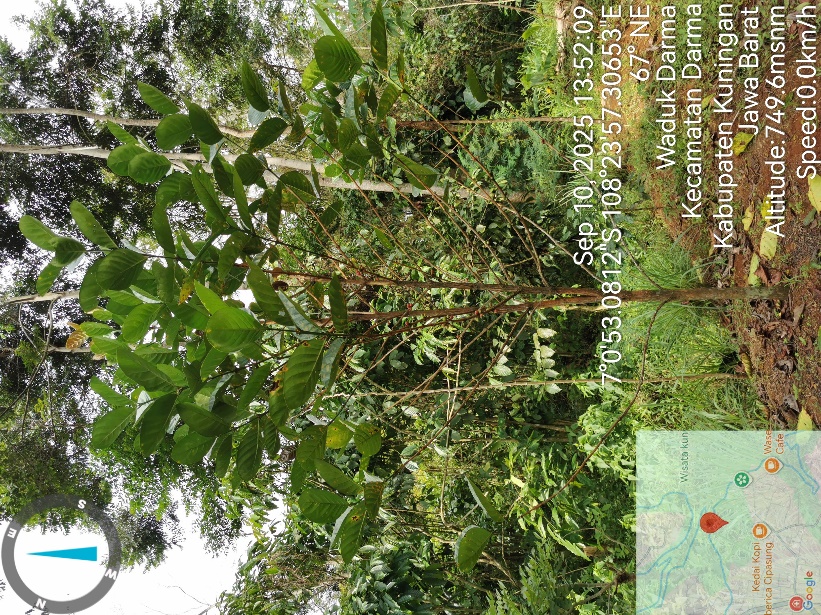 | 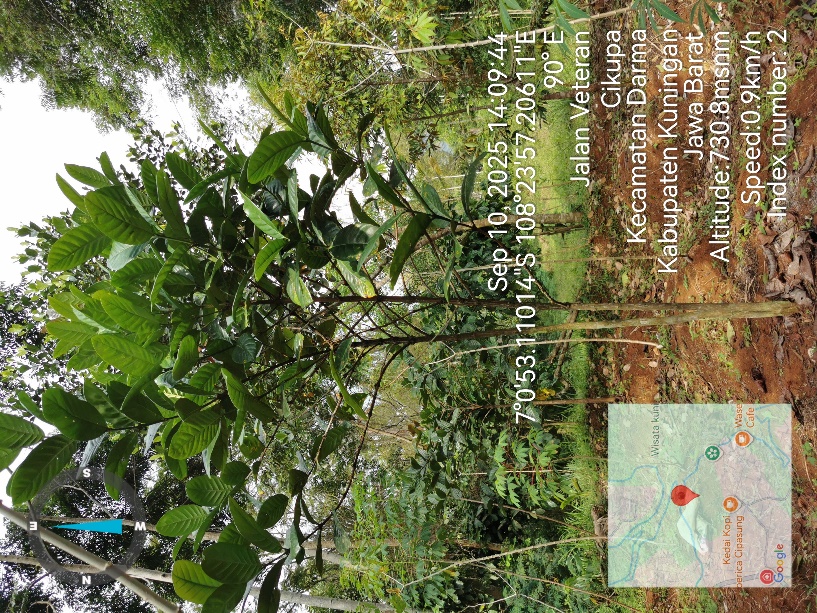 |
